# Black Silicon for Solar Fuels

**DOI:** 10.1002/advs.78342

**Published:** 2026-10-20

**Authors:** Neul Ha, Kaiying Wang, Wooseok Yang

**Affiliations:** ^1^ School of Chemical Engineering Sungkyunkwan University Suwon Republic of Korea; ^2^ Department of Microsystems University of South‐Eastern Norway Horten Norway; ^3^ SKKU Institute of Energy Science and Technology (SIEST) Sungkyunkwan University Suwon Republic of Korea; ^4^ Department of Future Energy Engineering (DFEE) Sungkyunkwan University (SKKU) Suwon Republic of Korea

**Keywords:** black silicon, photoelectrochemical, solar to fuel

## Abstract

Photoelectrochemical (PEC) solar energy conversion offers a promising route for transforming sunlight into chemical fuels and value‐added products through light‐driven redox reactions at semiconductor–electrolyte interfaces. Among semiconductor photoabsorbers, black silicon (b‐Si) is particularly attractive because its micro‐/nanostructured surface enables ultralow reflectance, broadband light absorption, a large electrode–electrolyte interfacial area, and versatile integration of catalysts and protective layers. These characteristics allow b‐Si to combine effective photon management with interfacial reaction engineering, making it relevant to a broad range of PEC processes. This review first introduces the fundamental characteristics of b‐Si and summarizes representative fabrication methods, with emphasis on the relationships among processing, morphology, and physicochemical properties. It then examines how these properties affect PEC water splitting, nitrate reduction, nitrogen reduction, and carbon dioxide reduction. Particular attention is given to the roles of surface morphology, junction design, passivation, catalyst integration, charge separation and transport, and the local reaction environment in governing activity, selectivity, and stability. Finally, the major limitations of b‐Si‐based PEC systems—including charge‐carrier recombination, photocorrosion, parasitic reactions, and mass‐transport constraints—are critically assessed. On this basis, future research directions are proposed for developing reaction‐adaptive b‐Si photoelectrodes capable of efficient, selective, durable, and practically relevant solar‐fuel and chemical production.

## Graphical abstract

Black silicon couples broadband light harvesting with high‐surface‐area catalytic interfaces, offering a versatile photoelectrode platform for solar‐fuel production. Reaction‐specific analysis highlights trade‐offs involving recombination, chemical instability, catalyst‐induced optical shielding, competing reactions, and mass transport. Passivation, conformal protection, spatial catalyst placement, selective interfaces, bubble management, and junction engineering define pathways toward efficient, selective, and durable conversion.

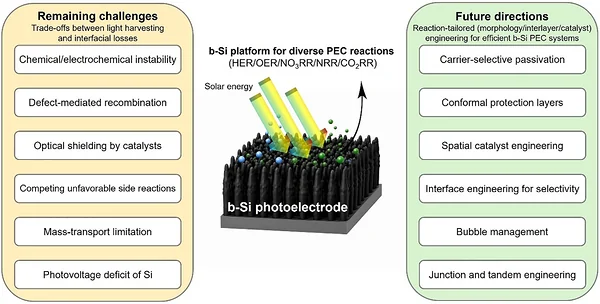

## 1 Introduction

Silicon (Si) has long been regarded as one of the most established semiconductors for solar energy conversion because of its suitable bandgap of about 1.12 eV, earth abundance, low toxicity, and mature processing technology. These advantages have made Si central to photovoltaic and photoelectrochemical energy systems [1, 2, 3, 4]. However, conventional Si still suffers from several intrinsic limitations for efficient light harvesting. Its surface reflectance is typically around 30%–40% over the visible‐to‐near‐infrared spectral range, resulting in substantial optical losses [5, 6, 7, 8]. In addition, because Si is an indirect‐bandgap semiconductor, its absorption coefficient decreases markedly near the band edge and in the near‐infrared region. Although the absorption coefficient can reach the order of 10^4^–10^5^ cm^−1^ at shorter visible wavelengths, it decreases to around 10^3^ cm^−1^ or lower near the band edge. This weak absorption of long‐wavelength photons generally requires thick absorber layers to ensure sufficient photon harvesting [8, 9, 10]. Furthermore, the photovoltaic and photoelectrochemical performance of Si is highly sensitive to carrier recombination caused by crystallographic defects, impurities and surface states. For this reason, single‐crystalline Si is often preferred over multicrystalline or defect‐rich Si, since grain boundaries and defect‐associated states act as recombination centers that shorten minority‐carrier lifetime and hinder carrier transport [11, 12]. To overcome these limitations, extensive efforts have been devoted to engineering Si surfaces into micro‐ and nanoscale architectures.

Black silicon (b‐Si) refers to Si with micro‐ and/or nanoscale surface textures that strongly suppress reflection and give the surface a black appearance [13, 14, 15]. Such textures can be produced by several approaches, including metal‐assisted chemical etching, wet or electrochemical etching, reactive ion etching, laser irradiation and plasma‐based processing. Depending on the fabrication route, b‐Si can exhibit pores, nanowires, cones, needles, pillars or hierarchical structures [16]. These architectures reduce reflectance by creating a gradual effective refractive‐index transition between air and Si [17]. They also promote multiple scattering and internal reflection, thereby increasing the effective optical path length inside the absorber. As a result, b‐Si can achieve broadband light trapping and highly efficient optical absorption over a wide spectral range.

The relevance of b‐Si extends beyond optical antireflection. Its high aspect ratio morphology provides an enlarged semiconductor/electrolyte interface and offers abundant space for catalyst deposition, protective layer coating and functional interlayer integration [18, 19]. These features are particularly important for photoelectrochemical (PEC) systems, where solar‐to‐fuel conversion depends on coupled processes of light absorption, charge separation, carrier transport, interfacial charge transfer and catalytic reaction kinetics. In PEC devices, a semiconductor photoelectrode generates electron‐hole pairs under illumination. The photogenerated charges then drive redox reactions at the electrode/electrolyte interface, such as hydrogen evolution, oxygen evolution, nitrate reduction, nitrogen reduction or carbon dioxide reduction [20, 21, 22]. In this context, b‐Si can serve not only as a light harvesting platform but also as a three‐dimensional scaffold for constructing integrated catalytic interfaces.

However, the same structural features that make b‐Si attractive also introduce challenges. The enlarged surface area can increase surface states and defect‐assisted recombination. High aspect ratio structures can complicate conformal passivation and may accelerate corrosion in aqueous electrolytes. Catalyst loading must also be optimized because excessive coverage can block incident light or generate additional recombination pathways. Therefore, efficient b‐Si‐based PEC photoelectrodes require coordinated control of morphology, junction formation, passivation, catalyst integration and mass transport. A simple reduction in reflectance is insufficient to ensure improved PEC performance.

Although several previous reviews have mainly addressed b‐Si fabrication strategies or its utilization in photovoltaics [16, 23, 24, 25, 26], the role of b‐Si in PEC systems has not yet been comprehensively examined from a reaction‐dependent perspective. Here, we provide a systematic overview of b‐Si for PEC applications, beginning with its definition, representative fabrication methods and structural features that govern light absorption, charge transport, surface reaction behavior and interfacial stability. While b‐Si may also be relevant to broader PEC transformations [27, 28, 29], its role has been more systematically investigated in water splitting, nitrate reduction, nitrogen reduction and CO_2_ reduction. Accordingly, this review focuses on these representative solar fuel generation reactions and moves beyond a descriptive summary of individual studies to establish a design‐oriented perspective on how b‐Si photoelectrodes should be tailored for each reaction context.

This perspective is particularly important because PEC performance is inherently reaction dependent. Water splitting, nitrate reduction, nitrogen reduction and CO_2_ reduction involve different energetic requirements, band alignments, reaction potentials, mechanistic pathways, kinetic barriers and competing side reactions. Their physicochemical environments also differ substantially in terms of reactants, products, gas‐phase involvement and mass transport constraints. These factors strongly influence interfacial reaction efficiency and overall device operation [20, 30, 31, 32, 33, 34]. Within this context, the large surface area and strong light trapping capability of b‐Si can provide significant advantages, but they can also intensify surface recombination, parasitic reactions, passivation difficulties and reactant/product transport limitations. Therefore, efficient b‐Si‐based PEC photoelectrodes cannot be achieved by using b‐Si alone. They require integrated design of light management, junction structures, surface protection, catalyst interfaces and local reaction environments. By categorizing recent advances in b‐Si‐based PEC systems according to reaction type and analyzing them from the coupled perspectives of energetics, interfacial kinetics and mass transport, this review aims to provide practical design insights for the rational development of efficient and durable b‐Si photoelectrodes. Finally, the remaining challenges and future research directions are discussed to clarify how b‐Si can be further advanced toward practical PEC solar‐fuel platforms.

## 2 Advanced Light‐Harvesting Platform, b‐Si

### 2.1 Definition of b‐Si

Conventional bare Si exhibits relatively high optical reflectance. In the visible region, planar Si commonly reflects approximately 30%–40% of incident light, which limits photon utilization at the surface and often requires a relatively thick absorber for sufficient light harvesting [7, 10]. Black silicon (b‐Si) refers to Si whose surface is textured with micro‐ and/or nanoscale features that strongly suppress reflection and enhance light trapping. Because of this reduced reflectance, the surface loses the shiny gray appearance of conventional Si and appears black to the naked eye [13, 14, 15].

### 2.2 Key Characteristics of b‐Si

As shown in Figure 1, b‐Si offers several characteristics that are particularly useful for PEC applications. The most representative feature is ultralow optical reflectance. In planar Si, the abrupt refractive‐index mismatch between air and Si causes substantial Fresnel reflection [35]. In contrast, the textured surface of b‐Si provides a gradual effective refractive‐index transition from air to bulk Si. This graded‐index profile allows incident photons to enter the absorber more efficiently and enables b‐Si to act as a broadband anti‐reflective interface [17, 36]. Depending on the fabrication route and structural design, b‐Si has been reported to exhibit optical reflectivity as low as approximately 1% over a broad wavelength range of 350–2250 nm, together with near‐complete absorption across a wide spectral region [37].

**FIGURE 1:**
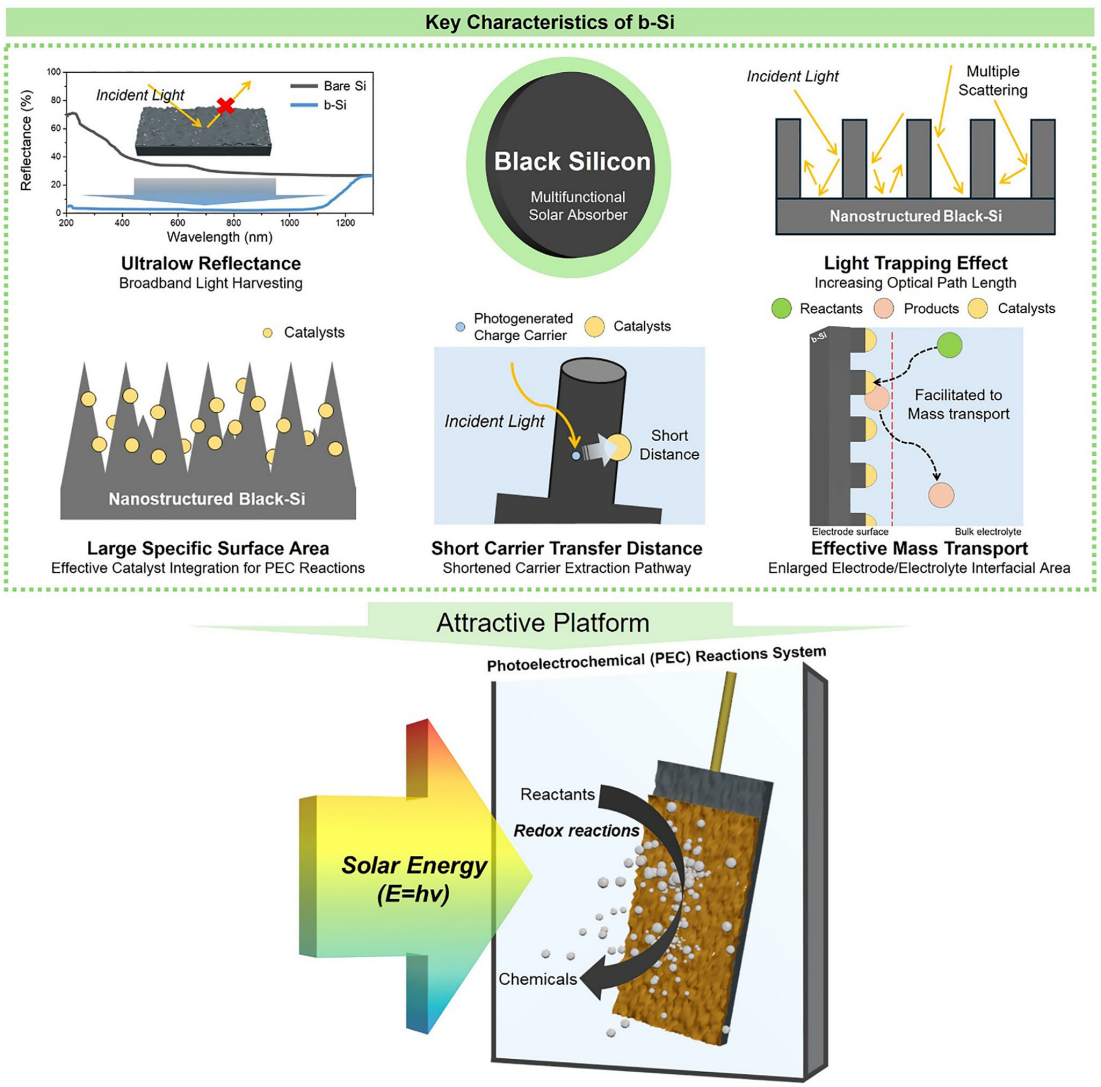
Schematic illustration of b‐Si as a multifunctional photoelectrode platform for photoelectrochemical solar‐fuel generation.

The micro‐/nanostructured morphology also provides strong light trapping. Once photons enter the textured layer, they undergo multiple scattering and internal reflection along pores, pillars, cones, or other high‐aspect‐ratio features. This diffuse propagation suppresses specular reflection and increases the effective optical path length within Si [38, 39]. The effect is especially important near the Si band edge, where absorption is intrinsically weak because of the indirect bandgap [40, 41]. In a simplified probabilistic description, if each light‐matter interaction provides an average absorption probability of (A), the cumulative absorption efficiency after (n) repeated scattering events can be expressed as

(1) $$\eta_{Abs}=1-{\left(1-A\right)}^{n}$$

This expression provides a conceptual illustration of how repeated scattering and multiple optical passes can enhance photon absorption by increasing the effective optical path length in textured semiconductor absorbers [42, 43]. For example, when (A = 0.60), the cumulative absorption reaches 84% after two interactions and approximately 99% after five interactions. Thus, the enhanced absorption of b‐Si mainly originates from photon management through reflection suppression and optical confinement rather than from a change in the intrinsic absorption coefficient or electronic band structure of Si.

Another important feature is hierarchical surface architecture. Many b‐Si structures combine micro‐ and nanoscale textures within a single surface. Microscale features redirect incident light and support multiple internal reflections, whereas nanoscale features strengthen the graded‐index effect. At the same time, the high‐aspect‐ratio three‐dimensional morphology enlarges the interfacial area relative to planar Si. This enlarged surface can provide more sites for catalyst loading, functional overlayer deposition and electrolyte contact [18, 19, 44, 45, 46].

These combined optical and interfacial features make b‐Si attractive for PEC systems, where efficient operation requires light absorption, charge separation, charge extraction and interfacial reaction kinetics to be optimized together. In well‐designed photoelectrodes, b‐Si can improve photon utilization while providing a favorable scaffold for catalyst integration and interfacial charge transfer. However, the same enlarged surface can also increase defect density and recombination if the surface is not properly passivated. Therefore, the practical value of b‐Si in PEC applications depends not only on light trapping but also on careful control of surface quality, catalyst coupling and charge‐transport pathways. Importantly, as summarized in Table 1, the relative importance of these optical, interfacial, and charge‐related characteristics varies with the target PEC reaction. For example, HER primarily requires efficient electron extraction and catalyst coupling, whereas OER places greater emphasis on hole transport and oxidative interface stabilization. These differences also extend to reaction kinetics and reactant availability. In HER, proton or water reactants are generally readily available in the electrolyte, and the relatively favorable reaction kinetics allow efficient electron delivery to be directly translated into catalytic turnover when suitable HER catalysts are employed. In contrast, OER involves intrinsically slower multistep kinetics; therefore, efficient hole delivery alone does not guarantee rapid oxygen evolution, and catalytic kinetics at the semiconductor–electrolyte interface become a critical limitation.

**TABLE 1:** Summary of reaction‐dependent roles and key interfacial/charge‐transfer requirements of b‐Si in PEC solar‐fuel conversion.

Reaction	Dominant role of b‐Si	Key interfacial/Charge‐related requirement
HER	Light harvesting and electron delivery	Efficient electron extraction, minimized surface recombination, and intimate coupling with HER catalysts
OER	Light harvesting and hole delivery	Efficient hole transport, suppressed interfacial recombination, and stable catalyst/protection‐layer integration
NO_3_RR	Photocarrier generation and high‐area catalyst support	Selective electron transfer to nitrate‐reduction sites, effective reactant access, and suppression of competing HER
NRR	Photocarrier supply and catalyst/mediator integration	Efficient electron delivery to N_2_‐activation sites, effective gas‐phase N_2_ supply and diffusion to the reaction interface, minimized recombination, and suppression of competing HER
CO_2_RR	Photocarrier supply and selective catalyst integration	Selective multielectron transfer to CO_2_RR sites, effective gas‐phase CO_2_ supply and diffusion to the reaction interface, efficient interfacial charge transfer, and suppression of competing proton reduction

For NRR and CO_2_RR, the limited availability of gaseous N_2_ and CO_2_ in aqueous electrolytes introduces an additional mass transport constraint. Accordingly, the effective utilization of the enlarged b‐Si surface requires not only efficient electron transfer but also sufficient reactant diffusion and accessibility to catalytic sites. The morphology should therefore provide a balance between high interfacial area and open transport pathways. Moreover, because these multielectron reactions involve coupled ionic and electronic processes, local reactant concentration, proton availability, and charge supply must be simultaneously regulated to suppress competing reactions and maintain selective conversion. In multielectron reduction reactions such as NO_3_RR, NRR, and CO_2_RR, selective interfacial electron transfer and suppression of competing reactions become increasingly important.

### 2.3 Fabrication Methods of b‐Si

As summarized in Figure 2, b‐Si can be fabricated through several representative texturing strategies. These methods are commonly divided into wet etching and dry etching routes. Wet etching relies on solution‐phase redox and dissolution reactions, whereas dry etching uses plasma‐assisted reactions, energetic ion bombardment, or laser‐induced melting and ablation. Because each method proceeds through a different physicochemical mechanism, it produces distinct surface morphologies and different levels of reflectance suppression, surface damage, scalability and process cost [16, 47]. The following sections summarize major wet routes including metal‐assisted chemical etching (MACE), wet chemical etching and electrochemical etching, followed by dry routes such as reactive ion etching (RIE), laser irradiation and plasma immersion ion implantation (PIII).

**FIGURE 2:**
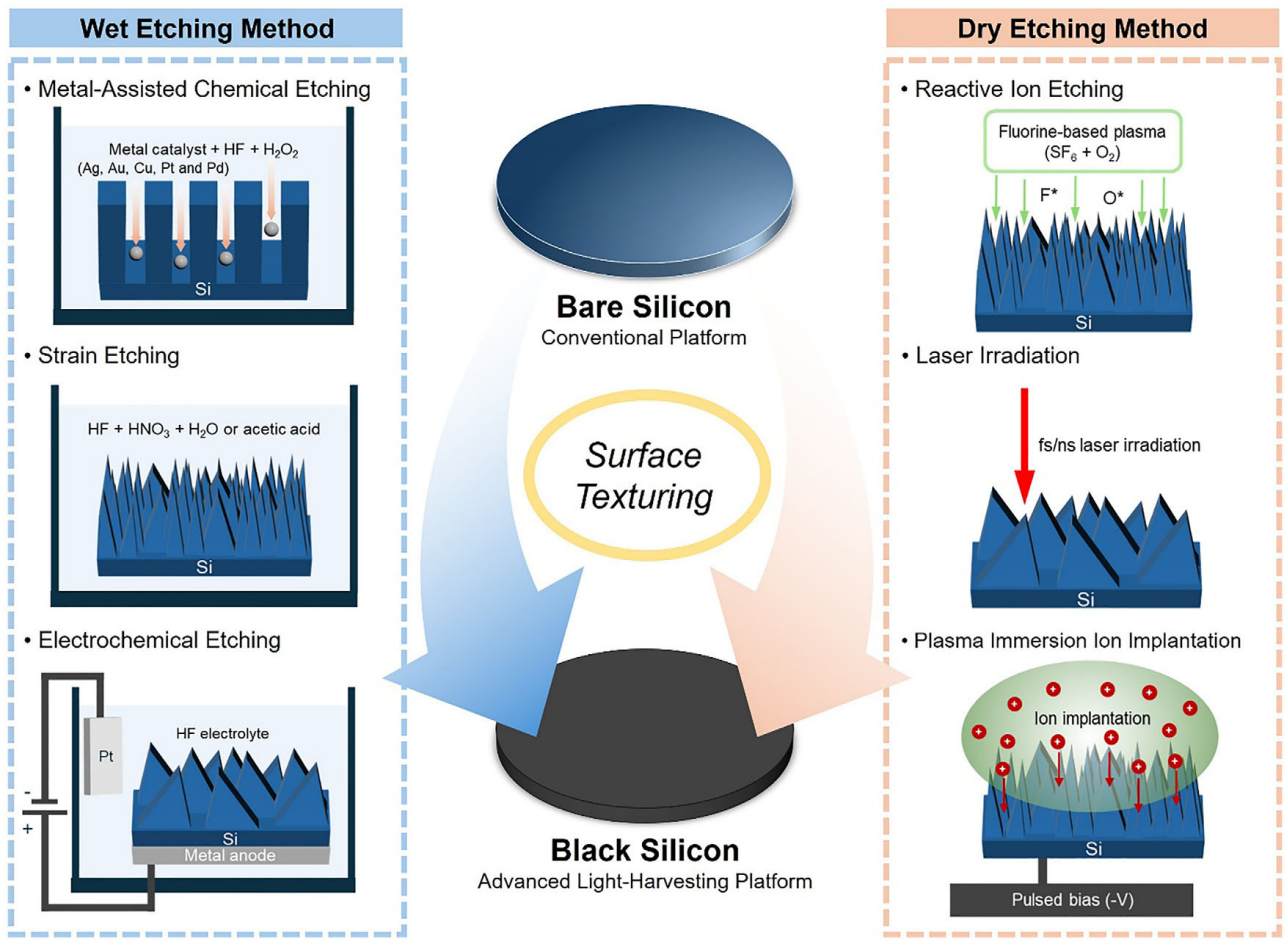
Schematic overview of representative wet and dry etching methods for b‐Si fabrication. Wet etching methods include MACE, stain/chemical etching and electrochemical etching, whereas dry etching methods include RIE, laser treatment and PIII.

#### 2.3.1 Metal‐Assisted Chemical Etching (MACE)

Metal‐assisted chemical etching is one of the most widely studied routes for b‐Si fabrication. In a typical MACE process, metal nanoparticles such as Ag [48, 49, 50], Au [51, 52, 53], Cu [54, 55], Pt [56, 57], or Pd [58] are deposited on the Si surface and then immersed in an HF‐based solution containing an oxidant, most commonly H_2_O_2_. The metal catalyst promotes oxidant reduction and injects holes into the underlying Si. The injected holes locally oxidize Si beneath the metal, while HF dissolves the oxidized Si species. These key interfacial steps can be schematically represented by the following reactions.

(2) $$H_{2}O_{2}+2H^{+}\to 2H_{2}O+2h^{+}$$

(3) $$Si+4HF_{2}^{-}+2h^{+}\to SiF_{6}^{2-}+2HF+H_{2}$$

In this process, H_2_O_2_ reduction occurs preferentially at the metal catalyst and generates holes that are transferred into the adjacent Si. The injected holes promote localized Si dissolution in the HF‐containing electrolyte, leading to preferential etching at the metal/Si interface [48, 59]. This coupled redox‐dissolution process enables anisotropic and directional texturing even though MACE is a wet chemical method. The final morphology is controlled by catalyst chemistry and geometry, oxidant‐to‐HF ratio, etching temperature and etching time. By tuning these parameters, MACE can generate nanowires, nanoholes, cones, inverted pyramids and other high aspect ratio textures [18, 60, 61, 62]. This morphological flexibility makes MACE useful for tailoring the optical response of b‐Si.

From a practical perspective, MACE is attractive because it is low cost, scalable and compatible with large area solution processing without vacuum‐based plasma equipment. Its limitations include possible metal contamination, the need for post‐etch catalyst removal, fluoride‐containing waste and sensitivity to local etching conditions. Therefore, reproducible b‐Si fabrication by MACE requires careful control of catalyst deposition, solution chemistry and cleaning procedures [59].

#### 2.3.2 Wet Chemical Etching

Wet chemical etching, especially stain etching, provides a simple metal‐free route to b‐Si. In this method, Si is exposed to acid mixtures such as HF/HNO_3_, sometimes with H_2_O or acetic acid to regulate the reaction rate and wetting behavior [53, 63, 64]. The process proceeds through coupled oxidation and dissolution. HNO_3_ oxidizes the Si surface, while HF dissolves the resulting oxide [65]. This repeated cycle produces porous and chemically roughened Si surfaces.

The resulting morphology is strongly affected by the balance between oxidation and oxide removal. Etchant composition, reaction time and substrate properties are therefore important variables [63]. A higher relative HF content generally favors pore formation through efficient oxide dissolution, whereas excessive oxidation can shift the process toward surface smoothing or polishing.

The main advantages of wet chemical etching are low cost, operational simplicity and compatibility with large‐area processing. It is also useful for multicrystalline Si, where conventional alkaline texturing is less effective because of randomly oriented grains [66]. However, wet chemical etching usually provides less precise morphology control than MACE, RIE or laser processing. It often produces porous or irregularly roughened layers rather than highly tunable high‐aspect‐ratio architectures. As etching proceeds, pore deepening may become limited by diffusion‐governed mass transport of reactants and products within the etched structure. In addition, hazardous acid handling and fluoride‐containing waste remain practical concerns. For these reasons, wet chemical etching is useful as an accessible texturing method, but it is less suitable when precise morphology control and highly optimized optical performance are required.

#### 2.3.3 Electrochemical Etching

Electrochemical etching is based on the anodic dissolution of Si in an electrolyte, typically an HF‐containing solution, under an externally applied bias or current [67, 68, 69]. Si acts as the anode, and its etched morphology is determined by applied potential or current density, electrolyte composition, temperature, etching time and wafer doping. Under appropriate conditions, the process can generate structurally complex Si surface textures [68].

Doping type and dopant concentration are particularly important because they influence carrier transport and pore formation. Illumination can also assist etching in n‐type Si by generating holes required for anodic dissolution [69, 70]. However, the same dependence on electrochemical and substrate parameters can limit reproducibility and large‐area uniformity. Scale‐up requires stable cell configuration and uniform current distribution across the substrate. Conventional electrochemical etching also relies on fluoride‐containing electrolytes, which introduces waste treatment and environmental issues. Thus, electrochemical etching is useful for controlled porous b‐Si fabrication, but it is generally less competitive than MACE or RIE for high‐throughput manufacturing that requires robust uniformity.

#### 2.3.4 Reactive Ion Etching (RIE)

Reactive ion etching is a representative dry etching method for fabricating anisotropic b‐Si textures. Inductively coupled plasma reactive ion etching (ICP‐RIE) is often used because it provides high plasma density and improved process controllability [16, 24]. In fluorine‐based plasmas such as SF_6_/O_2_, SF_6_ is dissociated into fluorine‐containing reactive species. Fluorine radicals react with Si to form volatile SiF_4_, while oxygen‐containing species contribute to the formation of a transient silicon oxyfluoride passivation layer on the surface and sidewalls. Ion bombardment locally removes or disrupts this passivating layer at exposed regions, allowing directional Si etching [71]. These plasma‐assisted dissociation, etching and passivation steps can be schematically represented as follows.

(4) $$SF_{6}\left(g\right)+e^{-}\to SF_{x}\left(g\right)+\left(6-x\right)F\cdot +e^{-}$$

(5) $$Si\left(s\right)+4F\cdot \;\to \;SiF_{4}\left(g\right)$$

(6) $$Si\left(s\right)+xO\cdot +yF\cdot \;\to \;SiO_{x}F_{y}\left(s\right)$$

The formation of volatile SiF_4_ promotes Si removal, whereas the transient SiO_x_F_y_‐rich layer suppresses lateral etching and assists anisotropic profile formation [72]. The dynamic balance between fluorine‐mediated etching, oxygen‐assisted passivation and ion‐induced surface activation produces sharp, irregular and high‐aspect‐ratio structures characteristic of b‐Si.

RIE‐based methods offer strong anisotropy and controllability. Gas composition, flow rate, chamber pressure, substrate bias, RF power, temperature and reactor configuration can be adjusted to form needle‐like features, rounded cones, aperiodic nanotip arrays or inverted pyramidal structures [73, 74]. ICP‐RIE is particularly useful because ion density and ion energy can be controlled more independently. This helps produce high‐aspect‐ratio textures with very low reflectance. Despite these advantages, RIE and ICP‐RIE require vacuum equipment and sophisticated plasma control, which increases cost and process complexity. Energetic ion bombardment can also introduce surface or subsurface damage that degrades carrier collection unless passivation or post‐treatment is applied [24, 75]. Depending on the plasma chemistry, fluorinated gases and byproducts may also raise environmental and safety concerns. Practical implementation therefore requires a balance between morphology control, surface quality, process cost and environmental burden.

#### 2.3.5 Laser Irradiation

Laser irradiation using femtosecond (fs) or nanosecond (ns) pulses is another route for producing b‐Si [76]. Laser pulses induce rapid local melting, ablation and resolidification of the Si surface. These nonequilibrium thermal processes generate micro‐/nanostructured textures such as conical spikes, microgrooves and ordered microstructured arrays [77, 78]. The final morphology depends on pulse duration, fluence, polarization, scan speed, pulse number and ambient environment. In reactive atmospheres, laser treatment can also modify the near‐surface composition and extend absorption into the near‐ or mid‐infrared region [79].

The strength of laser processing lies in its structural versatility and weak dependence on Si crystallographic orientation [16, 80]. It can produce designed optical responses and highly ordered surface architectures by controlling irradiation conditions. However, laser processing generally has lower throughput and relatively higher equipment cost than wet etching. Strong local thermal input can also create structural disorder, residual defects, and material damage [81, 82]. Therefore, laser irradiation is a powerful method for morphology design, but it is less favorable for low‐cost high‐volume manufacturing.

#### 2.3.6 Plasma Immersion Ion Implantation (PIII)

Plasma immersion ion implantation is a dry plasma‐based method that can texture Si while also modifying the near‐surface composition. During PIII, the Si substrate is immersed in plasma and subjected to pulsed biasing, which accelerates reactive ions toward the surface [16, 83, 84]. In fluorine‐containing plasmas, fluorinated species etch Si while a partially passivating Si_x_O_y_F_z_ layer can form. Pulsed ion bombardment repeatedly disrupts this transient layer and enables localized Si removal. Through this repeated etching‐passivation‐reactivation cycle, PIII can generate porous or needle‐like b‐Si textures [85, 86].

A distinctive feature of PIII is the possibility of simultaneous surface texturing and impurity incorporation. Depending on plasma composition and process conditions, it can tune both surface morphology and near‐surface doping or chemical composition [86]. This provides additional flexibility for controlling optical absorption. However, PIII is less commonly used than RIE or ICP‐RIE for mainstream b‐Si fabrication. It requires specialized plasma equipment and careful control of plasma chemistry, pulse bias and ion energy. Ion bombardment can also cause surface or subsurface damage, and fluorinated plasma chemistries raise environmental and safety issues. Accordingly, PIII is best regarded as a specialized route for combined texturing and functional modification rather than a general manufacturing method.

## 3 Photoelectrochemical Solar Energy Conversion Applications

To date, b‐Si has been widely explored as a promising platform for solar‐driven PEC conversion, owing to its micro‐/nanostructured surface that enables superior light absorption, enlarged interfacial area and abundant accessible reaction sites. Owing to these attributes, b‐Si has been applied to a broad spectrum of PEC reactions including hydrogen evolution, oxygen evolution, nitrate reduction, nitrogen reduction and CO_2_ reduction reaction. Nevertheless, these reactions differ markedly in their thermodynamic potential, band edge alignment requirements, interfacial charge transfer pathways, reaction kinetics and product selectivity [21, 22]. Therefore, the relative contributions of light harvesting, interfacial engineering and charge transport must be tailored to the specific requirements of each target reaction. In addition, although the textured morphology of b‐Si is beneficial for light management, the enlarged surface area may simultaneously aggravate surface defect formation and carrier recombination, thereby necessitating suitable passivation and catalytic modification. Furthermore, in PEC reactions involving gaseous products or intermediates, bubble generation at the electrode/electrolyte interface can alter local mass transport and active‐site accessibility, which in turn affects the measured photoelectrochemical performance [33]. In the following sections, selected recent studies are discussed to highlight the roles of b‐Si in different PEC reactions and to summarize key design strategies for b‐Si‐based photoelectrodes.

### 3.1 Hydrogen Evolution Reactions

Among the photoelectrochemical reactions investigated with b‐Si, the hydrogen evolution reaction (HER) has been the most extensively studied for b‐Si photocathodes because it directly converts photogenerated electrons into H_2_ through proton reduction. In PEC water splitting, HER occurs at the photocathode surface and is generally more kinetically accessible than the oxygen evolution reaction because it involves a two‐electron pathway rather than a four‐electron water‐oxidation process [22, 87]. For this reason, HER has frequently served as a benchmark reaction for evaluating newly developed Si‐based photocathode architectures. In this context, b‐Si provides important structural benefits including suppressed optical reflection, broadband light absorption and enlarged interfacial area for catalyst integration. However, its textured surface can also introduce defect states and interfacial recombination centers. Efficient b‐Si‐based HER photocathodes therefore require balanced control of light management, surface passivation, catalytic activity and junction engineering.

An early demonstration of b‐Si for PEC‐HER employed nanoporous b‐Si directly interfaced with an aqueous electrolyte without a dedicated HER cocatalyst, passivation layer, or buried junction [18]. The b‐Si photocathode was fabricated by metal‐assisted chemical etching and consisted of conical nanopores with diameters of approximately 20 nm and depths up to 590 nm. This nanoporous architecture formed a density‐graded antireflective layer and increased the effective surface area by about tenfold. As a result, the electrode showed broadband antireflection with reflectance below 2% in the ultraviolet and near‐infrared regions. The limiting photocurrent increased from about 30 mA cm^−2^ for polished Si to about 36 mA cm^−2^ at −1.0 V vs Ag/AgCl, and the onset potential shifted anodically by approximately 70 mV. These improvements demonstrate that morphology engineering alone can enhance photon harvesting and interfacial reaction area. At the same time, the increased dark current relative to planar Si revealed a central trade‐off of b‐Si photocathodes. The enlarged interface can promote HER kinetics, but it can also increase surface recombination through defective regions. Thus, the structural advantages of b‐Si are effective only when optical gain and interfacial charge loss are properly balanced.

Catalyst loading has been widely used to improve HER kinetics at the b‐Si/electrolyte interface. Pt remains the benchmark HER catalyst because its hydrogen adsorption free energy is close to thermoneutral [88]. When integrated with b‐Si, Pt lowers the interfacial overpotential and accelerates electron consumption for proton reduction. Zhao et al. reported oxidatively stable Pt‐modified nanoporous b‐Si photocathodes, in which Pt NPs were buried into the nanoporous framework through a secondary Pt‐assisted etching process [89]. This buried Pt configuration maintained intimate catalyst/Si contact even after surface oxidation. As a result, the onset voltage shifted anodically from about +0.25 to +0.40 V vs RHE, and the charge‐transfer resistance decreased without observable performance degradation for at least two months. This example shows that Pt can contribute not only to HER catalysis but also to long‐term performance retention when the catalyst/Si interface is structurally stabilized. A complementary strategy is found in Pt‐loaded sinuous b‐Si photocathodes, where the Si scaffold was engineered into a twisted nanoporous architecture rather than a conventional columnar texture [90]. This morphology improved HER stability across acidic, neutral and alkaline electrolytes by limiting oxide growth within the Si framework and preserving the catalyst/Si interface during operation. This example indicates that long‐term Pt/b‐Si performance can be improved not only by stabilizing the buried catalyst contact but also by controlling the intrinsic geometry of the b‐Si scaffold.

To reduce reliance on noble metals [18, 89, 90, 91], transition‐metal phosphides have been introduced as alternative HER catalysts. Cobalt phosphide is particularly attractive because phosphidation can tune the electronic structure of Co and provide more favorable hydrogen adsorption/desorption energetics [92]. When coupled with structured Si photocathodes, CoP_x_ catalysts can complement the optical and carrier collection advantages of b‐Si. Thalluri et al. developed an inverted pyramid p‐Si photocathode coated with a thickness‐gradient Co_2_P layer [93]. In this design, Co and P sites functioned cooperatively as hydride‐ and proton‐accepting centers, while the thickness gradient helped reduce parasitic light absorption. The SiIP@Co_2_P photocathode delivered 35.2 mA cm^−2^ at 0 V vs RHE and sustained HER currents above 30 mA cm^−2^ for at least 150 h. The Co_2_P layer therefore acted as both a HER catalyst and a protective interface. A related CoP_x_/Si nanowire photocathode showed a low HER overpotential of about −0.25 V vs RHE, a positive potential shift of +0.34 V and a hydrogen yield rate of 172.79 µmol cm^−2^ h^−1^ during 8 h operation [94]. These improvements were attributed to enhanced charge separation, reduced charge‐transfer resistance and increased exposure of HER‐active sites.

Non‐noble metal chalcogenides have also been explored because of their low cost, compositional tunability and edge‐rich catalytic structures. MoS_x_ is representative in this category. MoS_x_ quantum dots on b‐Si provided abundant HER‐active sites and intimate semiconductor/catalyst contact [98]. The MoS_x_/b‐Si photocathode showed an onset potential of 0.255 V vs RHE, a photocurrent density of 12.2 mA cm^−2^ at 0 V vs RHE and a hydrogen evolution rate of 226.5 µmol h^−1^ cm^−2^. The improvement originated from accelerated interfacial electron transfer, increased catalytic site density, favorable band bending and partial surface protection. As shown in Figure 3a, an amorphous MoS_x_ layer grown on b‐Si by atomic layer deposition further demonstrated the dual function of chalcogenide coatings [45]. A conformal 3 nm MoS_x_ film served simultaneously as a HER catalyst and a passivation layer. Owing to its S‐deficient metallic character and lower work function, the amorphous MoS_x_ layer improved HER kinetics and enhanced band bending at the Si interface. The optimized photocathode achieved an onset potential of 0.27 V vs RHE at 1 mA cm^−2^, a kinetic overpotential of about 0.2 V at 10 mA cm^−2^ and stable operation for more than 50 h while retaining at least 87% of its initial performance.

**FIGURE 3:**
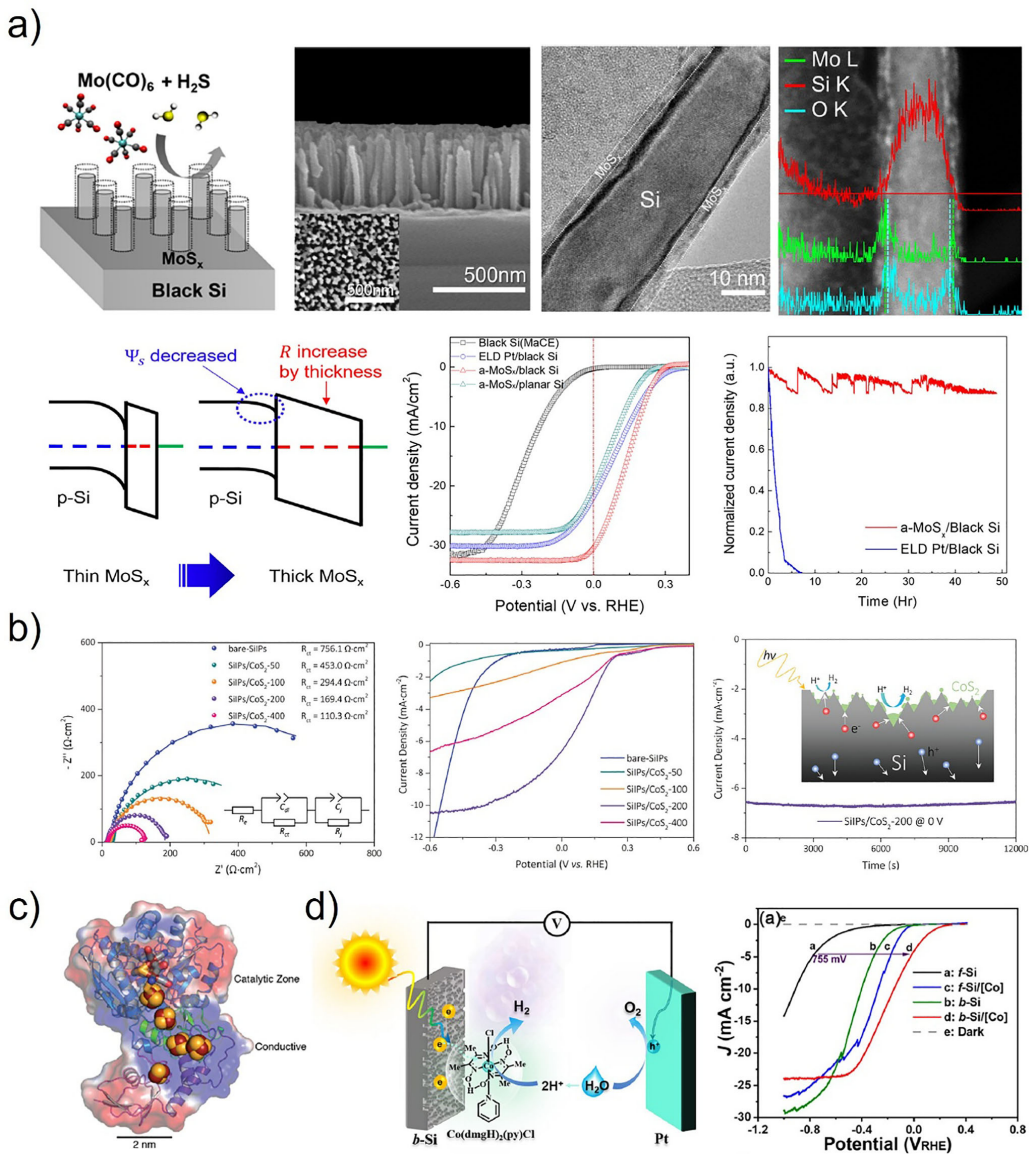
Representative b‐Si‐based photocathodes for PEC‐HER using non‐noble metal and molecular catalysts. (a) ALD‐grown amorphous MoS_x_‐coated b‐Si photocathode. SEM, HRTEM, EDS line‐scan, band diagrams, LSV curves and stability data show conformal MoS_x_ coating, thickness‐dependent junction behavior and improved HER activity/stability relative to unmodified b‐Si. Reproduced with permission [45]. Copyright 2022, American Chemical Society. (b) CoS_2_‐modified inverted‐pyramid b‐Si photocathode. Nyquist plots, J–V curves and stability data show reduced interfacial charge‐transfer resistance and enhanced HER performance after optimized CoS_2_ loading. Adapted with permission [95]. Copyright 2020, Royal Society of Chemistry. (c) Structural model of [FeFe]‐hydrogenase, highlighting catalytic and conductive domains for bio‐assisted proton reduction. Adapted with permission [96]. Copyright 2016, American Chemical Society. (d) Molecular Co catalyst‐modified nanoporous b‐Si photocathode. The device schematic and J–V curves show that nanoporous b‐Si and Co(dmgH)_2_(py)Cl jointly enhance light harvesting, charge transfer and HER kinetics. Adapted with permission [97]. Copyright 2023, Royal Society of Chemistry.

CoS_2_‐modified inverted‐pyramid b‐Si photocathodes provide another example of chalcogenide‐assisted HER. The inverted‐pyramid architecture was designed to mitigate some drawbacks of dense porous b‐Si by combining low reflectance with improved minority carrier lifetime and quasi‐hydrophilic behavior [95]. These properties are beneficial for proton transport and bubble release during HER. As shown in Figure 3b, the Nyquist plots and J–V curves reveal that optimized CoS_2_ loading substantially reduces interfacial charge transfer resistance and enhances the HER response of the SiIP photocathode under simulated solar illumination. After CoS_2_ loading, the charge‐transfer resistance decreased from 756.1 to 169.4 Ω cm^2^, indicating faster interfacial electron transfer. The optimized SiIP/CoS_2_‐200 photocathode exhibited an onset potential of 0.22 V vs RHE, a photocurrent density of 6.60 mA cm^−2^ at 0 V vs RHE and a saturated photocurrent density of 10.4 mA cm^−2^ at −0.45 V vs RHE. It also maintained a stable photocurrent of about 6.60 mA cm^−2^ for 12 000 s in acidic electrolyte. The performance enhancement can be attributed to the combined effect of catalytically active CoS_2_ and the Si/CoS_2_ heterojunction, which promotes band bending, suppresses recombination and accelerates electron extraction.

In addition to inorganic catalysts, molecular and biological catalysts have been integrated with nanoporous b‐Si. As illustrated in Figure 3c, [FeFe]‐hydrogenase‐modified b‐Si photocathodes demonstrated that a large‐area nanostructured Si scaffold can electrically couple with biological HER catalysts [96]. The resulting biohybrid photoelectrode exhibited improved onset potential and photocurrent density relative to bare b‐Si, mainly due to reduced interfacial charge‐transfer resistance and enzyme‐mediated proton reduction. Xie et al. further immobilized the cobaloxime‐type molecular catalyst Co(dmgH)_2_(py)Cl on b‐Si [97]. The b‐Si/[Co] photocathode in Figure 3d delivered 4.15 mA cm^−2^ at 0 V vs RHE, an onset potential of 0.31 V vs RHE and a maximum ABPE of 0.17%. Compared with planar Si, the nanoporous b‐Si scaffold provided a larger electrochemical surface area for molecular catalyst loading. The Co complex then accelerated proton reduction through its well‐defined catalytic center. These findings show that b‐Si can serve as a photoabsorber with large surface area not only for inorganic cocatalysts but also for molecularly defined HER active sites.

Passivation layers are equally important for b‐Si photocathodes because they suppress surface recombination and protect Si from electrolyte‐induced degradation. Oxide layers are widely used because they can provide chemical stability, optical transparency and conformal coverage on complex nanostructures [21, 99, 100]. They can also modify band bending, improve catalyst dispersion and act as electronically selective interlayers when properly designed. Zheng et al. reported a Pd‐decorated b‐Si photocathode protected by crystalline TiO_2_ with graded oxygen defects [101]. Unlike amorphous oxide coatings that may impede charge transport, the crystalline TiO_2_ layer provided a dense corrosion barrier, while the oxygen‐defect gradient created conductive pathways for electron transport toward Pd catalytic sites. As shown in Figure 4a, the Pd/b2‐TiO_2_/b‐Si photocathode achieved an onset potential of 0.32 V vs RHE, a saturated photocurrent density of about 35.3 mA cm^−2^ and stability for over 100 h at 10 mA cm^−2^ in 1.0 m NaOH. This result illustrates that protection layers can simultaneously improve durability and charge transport when their defect structure is rationally engineered.

**FIGURE 4:**
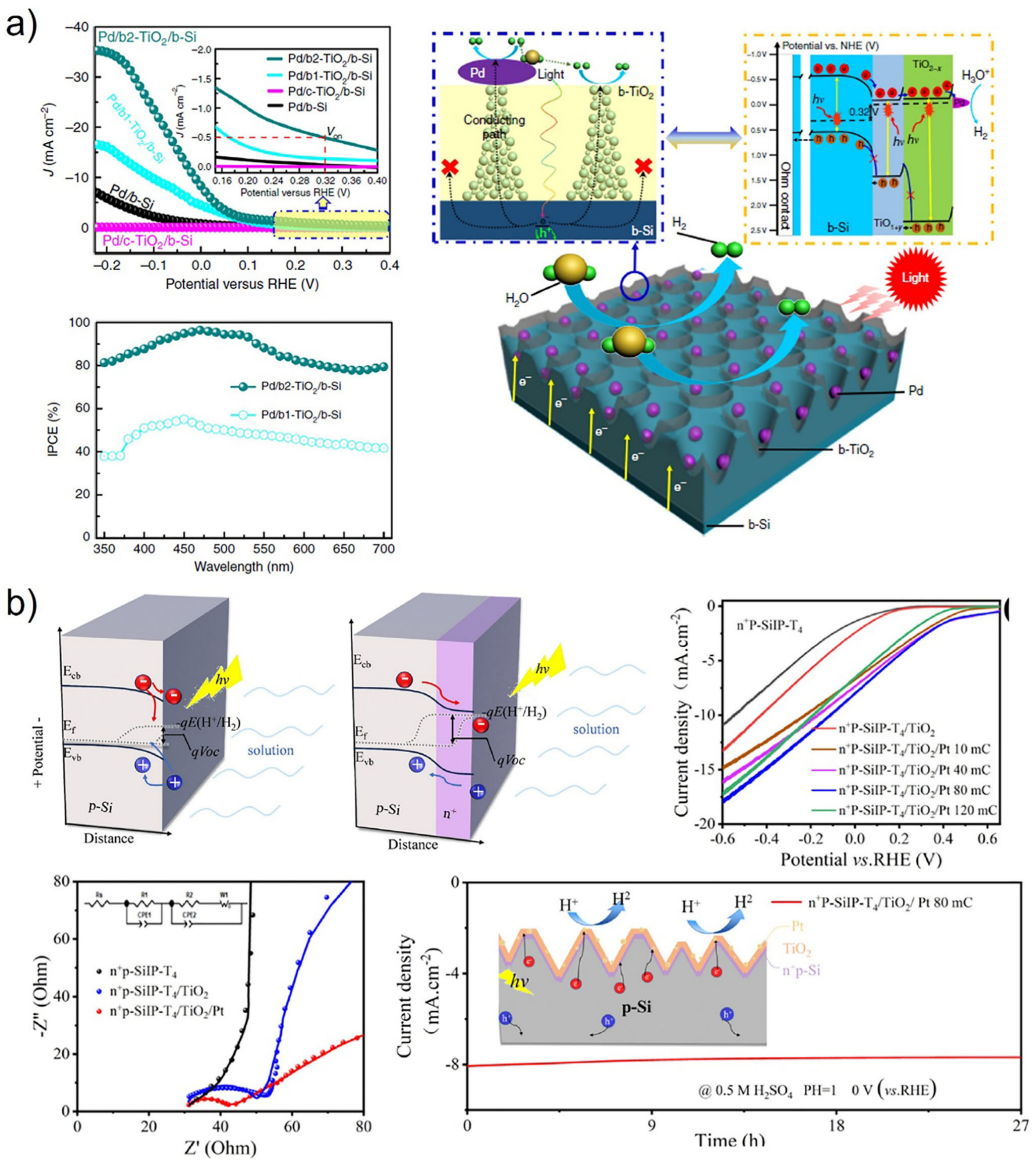
(a) Pd‐decorated b‐TiO_2_‐protected b‐Si photocathode. J–V curves compare Pd/b‐Si, Pd/c‐TiO_2_/b‐Si, Pd/b1‐TiO_2_/b‐Si and Pd/b2‐TiO_2_/b‐Si in 1.0 m NaOH under 1 sun illumination, showing the enhanced HER activity of Pd/b2‐TiO_2_/b‐Si. The IPCE spectra measured at −0.22 V vs. RHE further indicate improved photon‐to‐current conversion after introducing graded oxygen defects into the crystalline TiO_2_ protection layer. The schematic illustrates electron transport through defect‐mediated conducting pathways in b‐TiO_2_, followed by hydrogen evolution on Pd catalytic sites. Reproduced with permission [101]. Copyright 2018, The Author(s), published by Springer Nature. (b) Laminated n^+^p‐SiIP/TiO_2_/Pt inverted pyramid b‐Si photocathode. Band diagrams compare p‐Si and n^+^p‐Si photocathodes in contact with the H^+^/H_2_ redox couple, highlighting enhanced band bending and charge separation after forming the buried n+p junction. J–V curves of Pt‐loaded n^+^p‐SiIP‐T_4_/TiO_2_ photocathodes show the effect of Pt loading on HER performance. Nyquist plots of SiIP‐based photocathodes measured at 0 V vs. RHE under 1 sun illumination reveal reduced interfacial charge transfer resistance after TiO_2_ and Pt integration. The chronoamperometric stability test demonstrates sustained hydrogen evolution of the optimized n^+^p‐SiIP‐T_4_/TiO_2_/Pt 80 mC photocathode in acidic electrolyte. Adapted with permission [102]. Copyright 2024, Elsevier B.V.

Further improvements have been achieved by combining oxide passivation with junction engineering. Lu et al. developed an n^+^p‐SiIP/TiO_2_/Pt photocathode based on inverted pyramid b‐Si, where a vertical n^+^–p junction was introduced by phosphorus ion implantation [102]. As shown in Figure 4b, the buried junction strengthened the built‐in electric field and promoted directional separation of photogenerated carriers. The inverted pyramid morphology enhanced light trapping, while TMAH treatment smoothed defect‐rich sidewalls and reduced recombination. A conformal ALD TiO_2_ layer provided chemical protection and interfacial passivation, and Pt NPs accelerated proton reduction. The optimized n^+^p‐SiIP/TiO_2_/Pt photocathode generated approximately 8.0 mA cm^−2^ at 0 V vs RHE and exhibited a relatively positive onset potential of 0.49 V vs RHE. More importantly, it maintained stable HER operation for over 27 h in acidic electrolyte, indicating that the combined junction, TiO_2_ passivation and Pt cocatalyst architecture improved both activity and operational durability. This architecture demonstrates that the performance of b‐Si photocathodes can be enhanced by combining morphology‐driven light trapping, buried‐junction charge separation and oxide passivation in a single device.

This interfacial design was further advanced using an ultrathin TiO_2_/HfO_2_ bilayer. In this configuration, TiO_2_ contributed to antireflection and passivation, whereas high‐κ HfO_2_ reinforced the interfacial electric field, improved chemical stability and promoted more uniform Pt NPs dispersion [103]. The resulting n^+^p‐SiIP1‐O_2_/TiO_2_/HfO_2_/Pt photocathode delivered −20.2 mA cm^−2^ at 0 V vs RHE, an onset potential of 0.52 V vs RHE and an ABPE of 2.73%. It retained about 65% of its initial current over 24 h in acidic electrolyte. The improved activity arose from the combined effects of the n^+^–p junction and bilayer passivation, which reduced interfacial defects, lowered charge transfer resistance, reinforced favorable band bending and stabilized the b‐Si/Pt interface during HER operation. Table 2 provides a comparative overview of the representative PEC‐HER performance and stability of b‐Si‐based photocathodes.

**TABLE 2:** Representative b‐Si‐based photocathodes for PEC‐HER.

Device structure	Electrolyte	Key performance	Stability	Ref.
SiIP@Co_2_P	0.5 m H_2_SO_4_	J = 35.2 mA cm^−2^ at 0 V vs RHE, V_onset_ = 0.38 V vs RHE	> 30 mA cm^−2^ for 150 h	[93]
n^+^p‐b‐Si/Co	1.0 m HClO_4_	J_sat_ = 32.6 mA cm^−2^, V_onset_ = 0.33 V vs RHE.	3 h at 1.0 V vs RHE	[104]
CoP_x_/b‐Si NW	0.5 m H_2_SO_4_	J_max_ ≈ 9.5 mA cm^−2^ at −0.25 V vs Ag/AgCl, V_10mA_ = −0.25 V vs RHE	8 h at −0.33 V vs RHE	[94]
MoS_x_ QD/b‐Si	0.5 m H_2_SO_4_ (pH 0.3)	J = 12.2 mA cm^−2^ at 0 V vs RHE, V_onset_ = 0.255 V vs RHE	8 h at 0 V vs RHE	[98]
ALD a‐MoS_x_/b‐Si	H_2_SO_4_ acidic electrolyte	J_sat_ = 32.6 mA cm^−2^ from LSV plateau, V_onset_ = 0.27 V vs RHE	>50 h, ≥87% retained	[45]
SnS_2_ QDs/b‐Si	0.5 m H_2_SO_4_ (pH 0.3)	J = 1.23 mA cm^−2^ at 0 V vs RHE, J_sat_ ≈ 41 mA cm^−2^ at −0.51 V vs RHE, V_onset_ = 0.235 V vs RHE	4 h at 0 V vs RHE	[105]
SiIP/CoS_2_‐200	H_2_SO_4_ (pH 1)	J = 6.60 mA cm^−2^ at 0 V vs RHE, J_sat_ = 10.4 mA cm^−2^ at −0.45 V vs RHE, V_onset_ = 0.22 V vs RHE	12000 s at 0 V vs RHE	[95]
[FeFe]‐hydrogenase/b‐Si	1.0 m phosphate buffer (pH 6.8)	J = 1.8–5.0 mA cm^−2^ at −0.6 V vs Ag/AgCl, V_onset_ = −0.22 V vs Ag/AgCl	5 h at 0.1 V vs RHE under 0.1 sun	[96]
Co(dmgH)2(py)Cl/b‐Si	0.5 m H_2_SO_4_	J = 4.15 mA cm^−2^ at 0 V vs RHE, V_onset_ = 0.31 V vs RHE	27 h at 1.0 V vs RHE	[97]
Pd/b2‐TiO_2_/b‐Si	1.0 m NaOH	J_sat_ ≈ 35.3 mA cm^−2^ from LSV plateau, V_onset_ = 0.32 V vs RHE	> 100 h at 10 mA cm^−2^	[101]
n^+^p‐SiIP‐T4/TiO_2_/Pt 80 mC	H_2_SO_4_ (pH 1)	J = 8.0 mA cm^−2^ at 0 V vs RHE, V_onset_ = 0.49 V vs RHE	> 27 h at 0 V vs RHE	[102]
n^+^p‐SiIP1‐O_2_/TiO_2_/HfO_2_/Pt	1.0 m H_2_SO_4_ (pH 1)	J = 20.2 mA cm^−2^ at 0 V vs RHE, V_onset_ = 0.52 V vs RHE	24 h continuous J–t test, ∼65% retained	[103]

***Note***: The reported stability values were obtained under different applied potentials or current densities, illumination conditions and testing protocols. Therefore, the stability durations should be interpreted as study‐specific results rather than directly comparable performance metrics.

### 3.2 Oxygen Evolution Reaction

Beyond their use as photocathodes for hydrogen evolution, b‐Si‐based photoelectrodes have also been investigated as photoanode platforms for oxygen evolution, the anodic half‐reaction in PEC water splitting. In this case, the design focus shifts from electron‐driven proton reduction at a cathodic interface to hole‐driven water oxidation at an anodic interface. This shift introduces distinct requirements because OER is a kinetically demanding four‐electron process that proceeds through multiple proton‐coupled electron‐transfer steps involving surface‐bound intermediates such as *OH, *O and *OOH. As a result, sluggish OER kinetics, efficient hole transport, appropriate valence‐band alignment and resistance to oxidative corrosion become central design considerations [20, 21]. Efficient b‐Si‐based OER photoanodes therefore require protective layers and water‐oxidation catalysts that can stabilize the Si surface, facilitate interfacial hole transfer and lower the overpotential associated with intermediate formation. These requirements make OER fundamentally distinct from HER and necessitate different catalyst‐integration and interfacial‐engineering strategies for b‐Si‐based photoelectrodes. Bare b‐Si alone generally provides insufficient OER activity and stability because the Si surface is susceptible to oxidation and lacks efficient catalytic sites for multistep water oxidation. Therefore, catalyst‐free b‐Si serves primarily as a baseline for evaluating the effects of protective layers, hole‐selective interfaces and OER catalysts.

This design principle was illustrated by a nanotextured n‐Si photoanode coated with mixed NiRuO_x_. In this architecture, chemically etched nanotextured Si served as the light absorbing scaffold, while sputtered NiRuO_x_ acted as a multifunctional interfacial layer [106]. The porous Si texture, with a thickness of approximately 680 nm, reduced specular reflection over the 350–1100 nm range and enlarged the accessible water‐oxidation interface. At the same time, the NiRuO_x_ layer suppressed the formation of insulating SiO_x_ under anodic water‐oxidation conditions and formed a Schottky contact that promoted charge separation. It also supported hole transport and supplied catalytic Ni/Ru sites for OER. However, this system also revealed a morphology‐dependent trade‐off. Deeper textures can enhance light trapping and enlarge the reaction area, but excessive texturing can increase surface defects and hinder conformal catalyst or protection layer coverage. The OER activity was mainly governed by the mixed‐valence NiRuO_x_ coating. Incorporating RuO_x_ into NiO_x_ increased the relative Ni(III) content, lowered film resistivity and improved water‐oxidation kinetics. As a result, the nanotextured b‐Si|NiRuO_x_ photoanode generated 1.34 mA cm^−2^ at 1.23 V vs RHE, with the photocurrent further increasing to 7.44 mA cm^−2^ at a 432 mV overpotential under 200 mW cm^−2^ illumination. Although gradual degradation was observed during bias cycling, this work established an important design principle for b‐Si OER photoanodes: optical texturing must be coupled with hole‐selective charge transport and catalytic protection under oxidative conditions.

A more explicit oxide passivation layer strategy was later demonstrated using conformal TiO_2_‐coated b‐Si photoanodes. Yu and Zhang et al. deposited an ultrathin amorphous TiO_2_ layer by ALD on nanoporous b‐Si and then introduced Co(OH)_2_ as the OER catalyst [19]. The b‐Si morphology consisted of densely arranged nanoporous channels with diameters of 25–40 nm and a depth of about 300 nm. This depth was chosen to improve light absorption while limiting recombination losses. The 8 nm TiO_2_ film conformally coated the channel surface without removing the light‐trapping geometry. Co(OH)_2_ was then distributed over the TiO_2_‐coated surface and partially infiltrated into the channels.

As shown in Figure 5a, the b‐Si/TiO_2_/Co(OH)_2_ photoanode produced a saturated photocurrent density of 32.3 mA cm^−2^ at 1.48 V vs RHE in 1 m NaOH. Its onset potential was approximately 1.16 V vs RHE. Importantly, the improved performance was not mainly due to additional light absorption because TiO_2_ did not significantly change the low reflectance of b‐Si. Instead, sulfite oxidation measurements indicated improved charge separation, with an efficiency of 86.1% for b‐Si/TiO_2_/Co(OH)_2_ compared with 79.6% for b‐Si/Co(OH)_2_. The TiO_2_ layer therefore acted as both a corrosion barrier and a surface‐defect passivation layer. It also extended the operational lifetime from less than 0.5 h for unprotected b‐Si/Co(OH)_2_ to 4 h while retaining 81.2% of the initial photocurrent. This result highlights the central role of conformal oxide passivation in b‐Si photoanodes, where water oxidation requires simultaneous light trapping, hole transport, catalyst integration and corrosion resistance.

**FIGURE 5:**
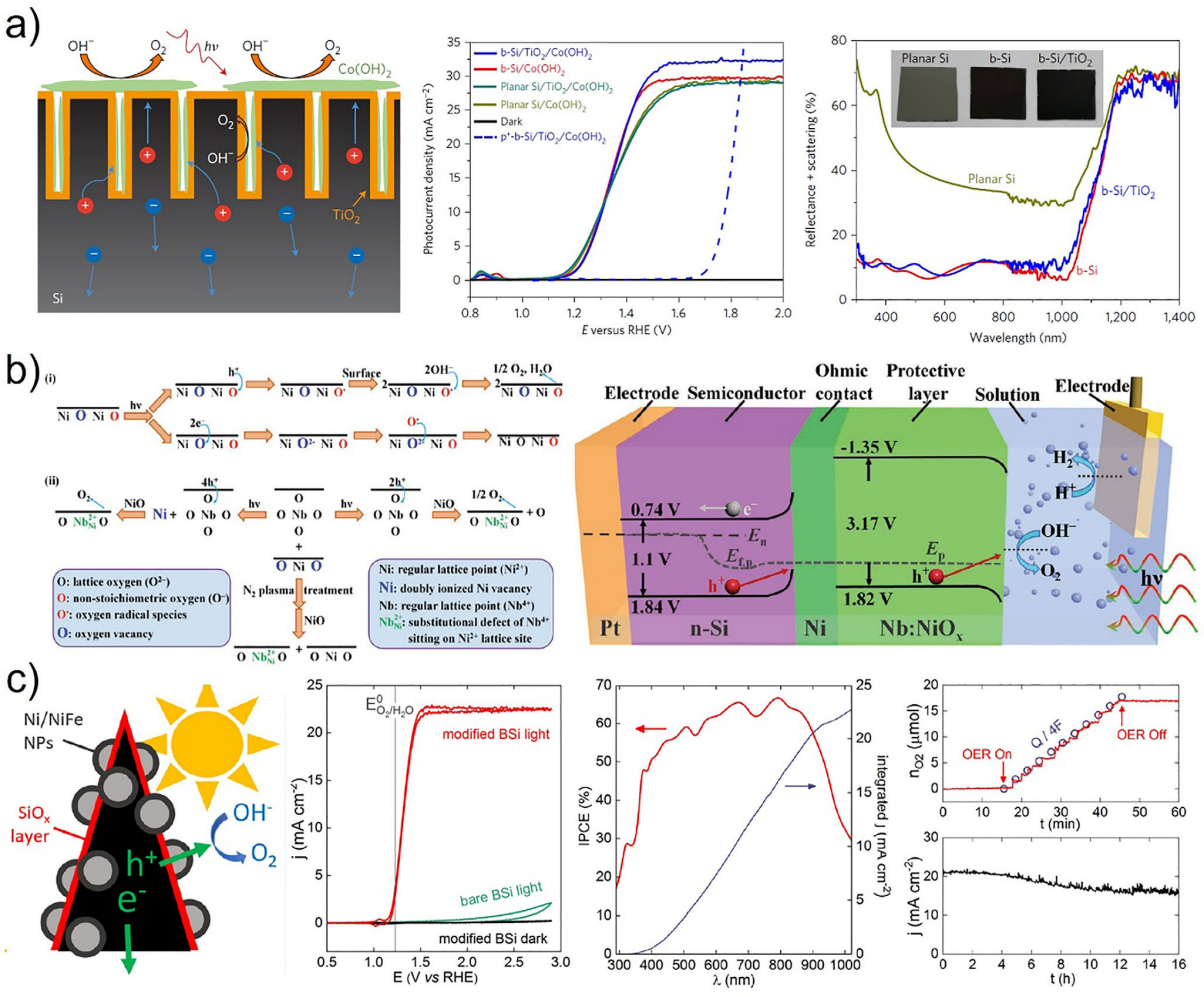
Representative examples of b‐Si‐based photoanodes for PEC‐OER. (a) b‐Si/TiO_2_/Co(OH)_2_ photoanode with a conformal TiO_2_ protection layer and Co(OH)_2_ OER catalyst. The schematic shows charge separation and oxygen evolution on the TiO_2_‐coated b‐Si architecture. J–V curves and reflectance/scattering spectra demonstrate enhanced photocurrent, strong light trapping and negligible optical loss after TiO_2_ coating. Adapted with permission [19]. Copyright 2017, The Author(s), published by Springer Nature. (b) Nb‐doped NiO_x_/Ni/b‐Si photoanode with defect‐mediated charge transport. The proposed mechanism and band diagram describe hole migration through NiO_x_‐related defect states, favorable band alignment for hole extraction and suppression of electron transfer to the electrolyte. Adapted with permission [107]. Copyright 2018, Wiley‐VCH GmbH. (c) Protection‐layer‐free Ni/NiFe‐modified b‐Si photoanode. Ni/NiFe nanoparticles on b‐Si form nanoscale Schottky junctions and OER‐active sites. J–V curves, IPCE, oxygen evolution and stability data demonstrate efficient and stable solar‐driven water oxidation. Reproduced with permission [108]. Copyright 2019, American Chemical Society.

Other TiO_2_‐based systems support the generality of this passivation concept. Si/TiO_2_ core–shell nanopillar photoanodes prepared by MACE and ALD used the TiO_2_ shell to protect the Si core and form an n‐Si/n‐TiO_2_ heterojunction [109]. Their PEC response depended strongly on nanopillar height and TiO_2_ thickness, showing that optical enhancement and charge transport must be optimized together. Plasma‐etched b‐Si coated with ALD TiO_2_ and CoO_x_ also showed improved photoanodic behavior during alkaline OER, with the conformal TiO_2_ layer helping to preserve the spiky b‐Si morphology [110]. These examples indicate that TiO_2_ is not merely a protective coating. It can also function as a charge‐regulating interlayer when its thickness, coverage and interface quality are properly controlled.

Beyond TiO_2_, defect‐engineered NiO_x_ has been explored as an electronically active protection layer. Zheng et al. reported a Nb‐doped NiO_x_/Ni/b‐Si photoanode in which b‐Si served as the light absorber, Ni provided electrical contact and the NiO_x_‐based overlayer acted as both a protection and a hole‐transport layer [107]. The upper region of the b‐Si texture was filled with the NiO_x_‐based layer, thereby isolating the unstable Si surface from alkaline electrolyte. The key feature of this design was that the oxide was not treated as a passive barrier. Instead, Nb doping and plasma treatment were used to regulate oxygen‐related defects in NiO_x_.

This defect regulation modified both carrier transport and interfacial energetics. Nb incorporation and plasma activation reduced detrimental oxygen vacancies while promoting Ni–O^−^‐related species that could assist hole migration. As illustrated in Figure 5b, the modified NiO_x_ layer produced more favorable band alignment with b‐Si. Its valence band facilitated hole transfer from Si into the oxide layer, while the conduction‐band barrier suppressed electron leakage toward the electrolyte. The optimized Nb‐doped NiO_x_/Ni/b‐Si photoanode reached approximately 29.1 mA cm^−2^ and a charge‐separation efficiency of about 81% at 1.23 V vs RHE. Since the samples showed comparable reflectance behavior, the improvement was mainly attributed to enhanced charge separation and transport rather than additional light harvesting. This work broadened the role of oxide protection layers from passive stabilization to active defect and band structure engineering.

Beyond protection layer engineering, b‐Si has also been used as a structured substrate for integrating secondary photoactive semiconductors. Here, b‐Si prepared by MACE was used as a low‐reflectance substrate for growing visible‐light responsive (Ga_1‐x_Zn_x_)(N_1‐x_O_x_) nanorods [111]. Unlike TiO_2_ or NiO_x_ passivation layers, the oxynitride nanorods acted as the photoactive component, while b‐Si provided light management, large surface area and improved nanorod adhesion. The nanoporous b‐Si substrate reduced reflectance from about 35% for single‐crystal Si to about 15%. The optimized nanorod electrode showed a photocurrent density of 55 µA cm^−2^ at 1.0 V, approximately 5.5 times higher than the corresponding single‐crystal Si‐based electrode. Although the absolute photocurrent was much lower than that of catalyst/protection‐layer‐based b‐Si OER photoanodes, this result showed that b‐Si can also serve as a structured platform for integrating visible light‐active semiconductor nanostructures. In contrast to continuous oxide passivation strategies, Oh et al. demonstrated a low‐cost protection layer‐free b‐Si photoanode based on selective Ni/NiFe nanoparticle deposition (Figure 5c) [108]. The n‐type b‐Si substrate was prepared by rapid wet chemical etching, producing a porous Si layer of approximately 10 µm thickness with vertically aligned macropores and spike‐like features. After Ni nanoparticles were electrodeposited on freshly hydrogenated b‐Si, a thin SiO_x_ layer was thermally formed on the exposed Si regions. This SiO_x_ layer did not act as a thick conformal protection layer. Instead, it guided the subsequent NiFe electrodeposition so that NiFe grew mainly on pre‐existing Ni NPs. The Ni core formed local Si/Ni Schottky junctions, while the NiFe shell provided OER‐active sites that could transform into NiFe oxyhydroxide species during operation.

This discontinuous nanoparticle architecture preserved much of the optical advantage of b‐Si. The visible‐range reflectance remained below 10% even after Ni/NiFe modification, indicating that severe optical blocking was avoided. Under AM 1.5G illumination in 1 m NaOH, the b‐Si/Ni/NiFe photoanode reached a maximum photocurrent density of 23.8 ± 0.8 mA cm^−2^ and produced O_2_ with a Faradaic efficiency of 96%. It also retained more than 75% of its initial photocurrent after 16 h. The result represents a distinct interfacial design route in which partial SiO_x_ formation, local Schottky junctions and discontinuous OER catalyst coverage are combined without relying on ALD‐processed conformal oxide protection.

Related protection layer‐free approaches further clarified the balance between catalytic coverage, light absorption and stability. Vertically aligned n‐Si microwire arrays decorated with electrodeposited Ni NPs showed that insufficient Ni coverage limits catalytic activity, whereas excessive Ni loading blocks incident light and lowers photocurrent [115]. With optimized Ni coverage, the n‐Si microwire/Ni photoanode reached a maximum photocurrent density of 35.5 mA cm^−2^ and retained approximately 90% of its initial current at 10 mA cm^−2^ after 6 h in alkaline electrolyte. This concept was extended to Fe NPs‐modified n‐Si photoanodes, as shown in Figure 6a [112]. Partially covered n‐Si/Fe photoanodes used Fe oxide/hydroxide surfaces for OER and an ultrathin SiO_x_ interlayer at the Si/Fe interface. In strongly alkaline electrolyte, higher Fe coverage improved durability but reduced photocurrent through optical blocking. Under milder K‐borate/Li‐borate buffer conditions at pH 9.6, the operation time exceeded 130 h. This behavior shows that catalyst coverage and electrolyte chemistry must be considered together when designing protection layer‐free Si photoanodes.

**FIGURE 6:**
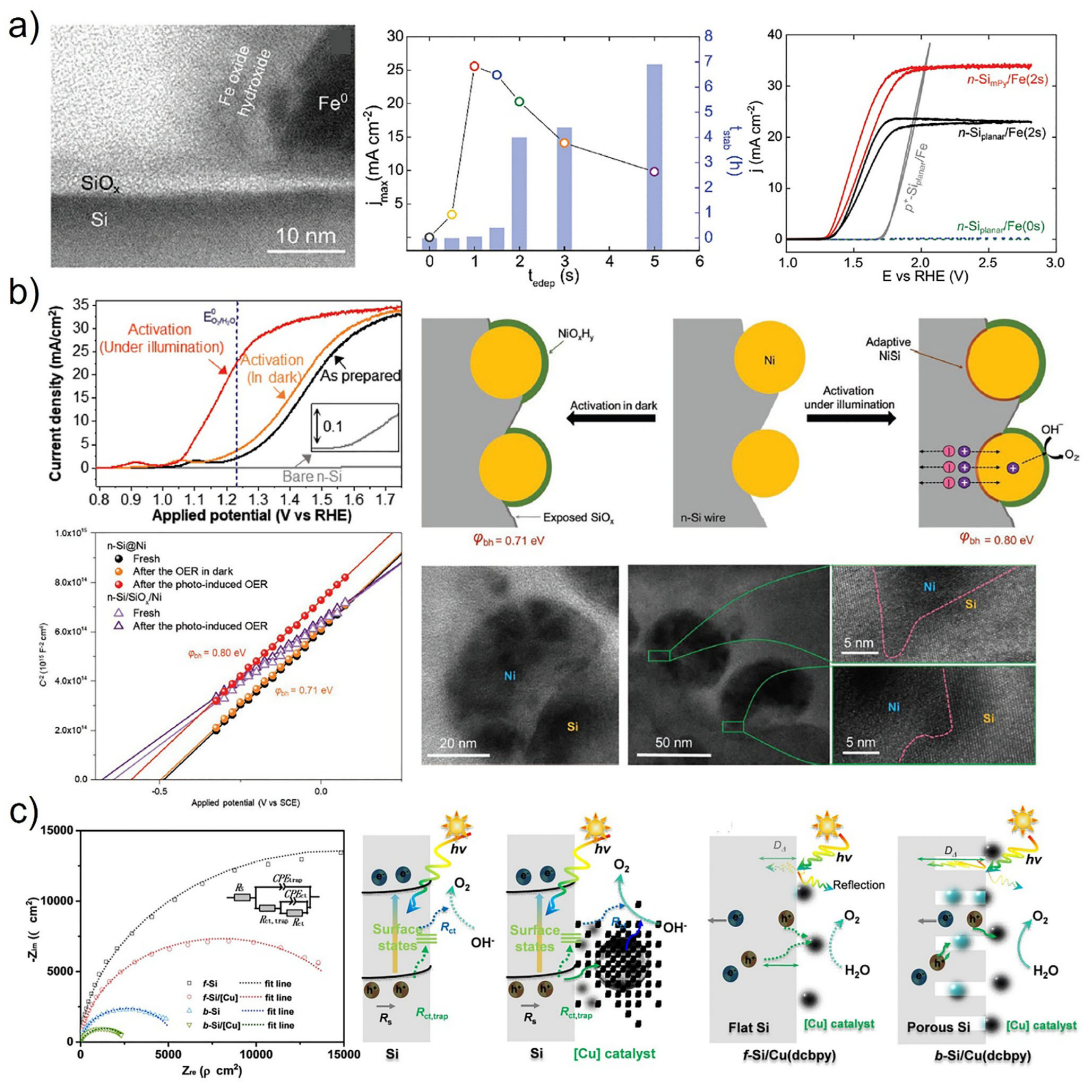
(a) n‐Si/SiO_x_/Fe photoanodes. Cross‐sectional HRTEM, electrodeposition‐time‐dependent photocurrent/stability data and CV curves reveal the optimized Fe coverage for balancing catalytic activity, light absorption and durability. Adapted with permission [112]. Copyright 2020, Wiley‐VCH GmbH. (b) Ni‐decorated porous n‐Si microwire photoanodes with light‐induced adaptive junctions. J–V curves, Mott–Schottky analysis, schematic models and HRTEM images show that photoactivation forms an adaptive NiSi interfacial phase, increases the barrier height and enhances charge separation. Reproduced with permission [113]. Copyright 2019, Wiley‐VCH GmbH. (c) Cu(dcbpy)‐modified b‐Si photoanodes. Nyquist plots and schematics demonstrate that nanoporous b‐Si improves water oxidation through light trapping, enlarged catalyst loading and reduced charge‐transfer resistance. Adapted with permission [114]. Copyright 2022, Wiley‐VCH GmbH.

The metal/Si junction concept was further advanced by identifying light‐induced adaptive interfacial reconstruction. Tung and Kuo et al. used porous Si microwire arrays with nanotextured surfaces and electrodeposited Ni nanoparticles to form b‐Si@Ni photoanodes [113]. Under illumination, a Ni redox response appeared before the OER onset, indicating that photogenerated holes from Si participated in the oxidation and restructuring of Ni species. After light‐induced activation, the potential required to reach 10 mA cm^−2^ decreased by 247 mV relative to the fresh photoanode, and the photocurrent at 1.23 V vs RHE increased by approximately six times. The activated electrode also showed stable operation for 1800 min at 1.25 V vs RHE. The main contribution of this study was the direct identification of an adaptive NiSi junction at the Ni/Si interface, as shown in Figure 6b. Under illuminated OER conditions, the interface reconstructed into an orthorhombic NiSi interlayer with an average thickness of about 4.5 nm. This transformation was not observed under dark activation, which suggests that photogenerated holes play a decisive role in driving interfacial reconstruction. The NiSi layer shifted the flat‐band potential in the negative direction and increased the effective Schottky barrier height from 0.71 to 0.80 eV. This stronger barrier promoted band bending, charge separation and hole transfer to the Ni oxide/oxyhydroxide catalytic surface. Thus, the enhanced OER performance originated not only from Ni catalysis but also from the formation of a light‐induced adaptive junction.

Molecular catalyst functionalization has also expanded the design space of b‐Si OER photoanodes. As shown in Figure 6c, a copper‐bipyridine molecular catalyst, Cu(dcbpy), was immobilized onto nanoporous b‐Si through chemisorption [114]. The nanoporous b‐Si surface had pores with diameters of approximately 5–10 nm and depths greater than 300 nm. This morphology improved light harvesting, increased catalyst loading and reduced interfacial charge‐transfer resistance compared with flat Si. The b‐Si/Cu(dcbpy) photoanode generated 6.31 mA cm^−2^ at 1.5 V vs RHE under AM 1.5G illumination at pH 11.0. It also exhibited a reduced Tafel slope and a lower overpotential for water oxidation than bare b‐Si. The pH‐dependent behavior was attributed to the monomer/dimer equilibrium of the Cu(dcbpy) catalyst. Dimeric hydroxyl‐complex species formed near pH 10–12 can promote O–O bond formation through a redox isomerization pathway, whereas monomeric Cu species are more closely associated with a water nucleophilic attack pathway. However, long‐term operation showed gradual photocurrent decay and XPS analysis indicated increased Si–O and O–Si signals. These observations suggest that molecular catalyst integration can improve OER kinetics, but additional protection remains necessary to suppress Si oxidation. Key PEC‐OER performance metrics of representative b‐Si‐based photoanodes, including current density and durability, are compiled in Table 3

**TABLE 3:** Representative b‐Si‐based photoanodes for PEC‐OER.

Device structure	Electrolyte	Key performance	Stability	Ref.
b‐Si/NiRuO_x_	N_2_ saturated 0.25 m Na_2_SO_4_ (pH 7.2)	J = 1.34 mA cm^−2^ at 1.23 V vs RHE, V_onset_ ≈ 0.98 V vs RHE	Noticeable degradation after 500 cycles.	[106]
p^+^n‐b‐Si/CoO_x_	1.0 m NaOH	J = 17 mA cm^−2^ at 1.23 V vs RHE, V_onset_ ≈ 1.05 V vs RHE	≥ 24 h	[116]
b‐Si/TiO_2_/Co(OH)_2_	1.0 m NaOH	J_sat_ = 32.3 mA cm^−2^ at 1.48 V vs RHE. V_onset_ ≈ 1.16 V vs RHE.	4 h, ∼81% retained	[19]
Nb:NiO_x_/Ni/b‐Si	1.0 m NaOH	J ≈ 29.1 mA cm^−2^ at 1.23 V vs RHE	—	[107]
b‐Si/Ni/NiFe	1.0 m NaOH	J_max_ = 23.8 ± 0.8 mA cm^−2^, FE_O2_ = 96%	16 h, ∼75% retained	[108]
b‐Si microwire/Ni	1.0 m KOH	J_sat_ = 35.5 mA cm^−2^ at ≈ 2.3 V vs RHE, V_onset_ = 1.08 V vs RHE	Stable operation at 10 mA cm^−2^ for several hours	[115]
b‐Si micropyramid/Fe	K‐borate/Li‐borate buffer (pH 9.6)	J_sat_ = 34 mA cm^−2^ at ≈ 1.8 V vs RHE, V_onset_ = 1.24 V vs RHE	> 130 h	[112]
p^+^n‐b‐Si/SnO_2_/W:BiO_4_/CoPi	0.1 m K‐Pi buffer (pH 7.0)	J ≈ 2.0 mA cm^−2^ at 1.23 V vs RHE, V_onset_ = −0.16 V vs RHE at 0.02 mA cm^−2^	18 h	[117]
b‐Si/Cu(dcbpy)	Mixed NaOH and Na_2_SO_4_ (pH 11.0)	J = 6.31 mA cm^−2^ at 1.5 V vs RHE	—	[114]
b‐Si/NiO/Cu(dcbpy)	1.0 m NaOH	J_sat_ = 16.63 mA cm^−2^ at 2.2 V vs RHE, V_onset_ = 1.13 V vs RHE	90 h at 2.2 V vs RHE	[118]

***Note***: The reported stability results were obtained under different applied potentials or current densities, illumination conditions and testing protocols. Therefore, these stability data should be interpreted as study‐specific results rather than directly comparable performance metrics.

### 3.3 Nitrate Reduction Reaction

PEC‐NO_3_RR has recently emerged as a promising solar‐assisted route for green ammonia production and nitrate remediation. Conventional ammonia synthesis is dominated by the Haber–Bosch process, which converts N_2_ and H_2_ into NH_3_ under high‐temperature and high‐pressure conditions. Although this process is highly mature and indispensable for fertilizer production, it requires substantial energy input and is closely associated with fossil fuel‐derived hydrogen [30, 119]. In this context, PEC‐NO_3_RR offers an alternative strategy because nitrate can serve as a soluble nitrogen source while water supplies protons for ammonia formation. This reaction therefore links solar‐assisted ammonia production with nitrate remediation and nitrogen resource recovery [120, 121].

Unlike HER and OER in water splitting, PEC‐NO_3_RR involves the selective multielectron reduction of dissolved NO_3_^−^ to NH_3_ or NH_4_^+^. Nitrate contains more labile N─O bonds than the N≡N bond in molecular N_2_, making it a more accessible nitrogen feedstock for ammonia formation. In a typical PEC‐NO_3_RR system, photogenerated electrons are transferred from the photocathode to catalytic sites, where NO_3_^−^ adsorption, deoxygenation and hydrogenation proceed through adsorbed intermediates such as *NO_3_, *NO_2_, *NO and *NH_x_. Meanwhile, photogenerated holes are consumed by an oxidation reaction at the counter electrode or photoanode. Selective ammonia formation remains challenging because NO_3_RR requires multiple electron‐ and proton‐transfer steps and competes strongly with HER. Depending on the catalyst surface and local reaction environment, partially reduced products such as NO_2_^−^, NO, N_2_O or N_2_ can also be generated [121, 122, 123, 124]. For b‐Si‐based PEC‐NO_3_RR, the design principle differs from that for water splitting. The textured Si architecture should function not only as a light absorber but also as a structured reaction interface for nitrate conversion. Its enlarged surface can accommodate catalytic sites and improve electrolyte accessibility, which can promote nitrate adsorption and interfacial electron transfer. At the same time, excessive surface area can intensify surface state recombination and parasitic HER if the interface is not properly passivated and catalytically regulated. Efficient b‐Si‐based PEC‐NO_3_RR therefore requires coordinated control of morphology, charge separation, surface protection and catalyst selectivity. The key objective is to direct photogenerated electrons toward nitrate‐to‐ammonia conversion rather than nonselective proton reduction. In the absence of a selective cocatalyst, however, b‐Si primarily functions as a light absorber and photocarrier source and generally lacks sufficient catalytic selectivity for multielectron nitrate‐to‐ammonia conversion. Catalyst‐free b‐Si therefore provides an important baseline for distinguishing the contribution of the b‐Si photoabsorber from that of subsequently introduced NO_3_RR catalysts. Because ammonia can be affected by background contamination and analytical artifacts, rigorous product validation is essential in PEC‐NO_3_RR. Appropriate blank/control experiments, such as measurements without nitrate or illumination, together with ^15^NO_3_^−^ isotope‐labeling tests, provide important evidence that the detected ammonia originates from nitrate reduction.

Au‐decorated ordered Si nanowire (NW) arrays provided an early example of how nanostructured Si can be engineered for selective PEC‐NO_3_RR [61]. The photoelectrode was fabricated from p‐type Si through a modified MACE process on a hexagonally patterned template, producing ordered Si NW arrays before the deposition of Au NPs. This ordered NW architecture improved mass transport and suppressed charge recombination compared with planar Si and randomly grown Si NWs. The O_SiNW/Au photocathode achieved a faradaic efficiency of 95.6% for NH_3_ production at 0.2 V vs RHE under simulated sunlight. This potential is more positive than the thermodynamic nitrate reduction potential, indicating that the Si photocathode supplied a useful photovoltage for reducing the external bias required for NO_3_^−^ conversion. Mechanistically, Au NPs promoted the kinetically demanding NO_3_^−^‐to‐NO_2_^−^ conversion step, while both Si and Au showed relatively weak activity toward competing water reduction. The ordered Si NW structure also enabled more uniform Au distribution without significantly compromising light absorption. These findings established that nanostructured Si can support selective PEC‐NO_3_RR when optical absorption, catalyst dispersion and HER suppression are controlled together.

Cu‐based b‐Si photocathodes have been further developed because Cu can promote nitrate adsorption and hydrogenation toward ammonium formation. Li et al. integrated Cu NPs with b‐Si NW arrays for acidic PEC‐NO_3_RR [50]. As shown in Figure 7a, the photocathode consisted of vertically aligned Si NWs uniformly decorated with Cu NPs. The HAADF‐STEM and elemental mapping images confirm homogeneous Cu loading along the Si NW surface, while the schematic illustrates electron transfer from Si to Cu active sites for NO_3_^−^‐to‐NH_4_^+^ conversion. The Si NWs suppress reflectance and provide a high‐area scaffold for catalyst loading, whereas Cu NPs serve as catalytic sites for nitrate activation in strongly acidic electrolyte. The optimized Cu–Si NW photocathode showed a more positive onset potential of about 0.3 V vs RHE and a saturated photocurrent density of approximately 34.29 mA cm^−2^ in 0.5 m H_2_SO_4_ containing nitrate under AM 1.5G illumination. The NH_4_^+^ faradaic efficiency reached 97.03% at −0.4 V vs RHE, and the NH_4_^+^ yield rate increased to 65.91 µmol h^−1^ cm^−2^ at −0.6 V vs RHE. In comparison, a catalyst‐free p‐Si control exhibited only limited NH_4_^+^ formation, with a maximum FE of 12.29% and a yield rate of 9.28 µmol h^−1^ cm^−2^ at −0.9 V vs RHE, highlighting the critical role of Cu decoration in promoting nitrate reduction. The NH_4_^+^ product was further validated using ion chromatography, ^1^H NMR, and ^15^NO_3_^−^ isotope‐labeling experiments, confirming that the detected ammonia originated from nitrate reduction. Together, these comparisons confirm the essential role of Cu decoration in promoting selective nitrate‐to‐ammonium conversion. These values were much higher than those of bare Si NWs, confirming the essential role of Cu in selective nitrate‐to‐ammonium conversion. The enhanced activity was attributed to the coupled effects of b‐Si NW light trapping and Cu‐mediated interfacial catalysis. The Cu/Si contact promoted charge separation and electron transfer to the catalyst surface, while Cu sites improved NO_3_^−^ adsorption and activation. The disappearance of transient photocurrent spikes after nitrate addition further suggested that photogenerated electrons were efficiently consumed by nitrate reduction rather than accumulating at the surface.

**FIGURE 7:**
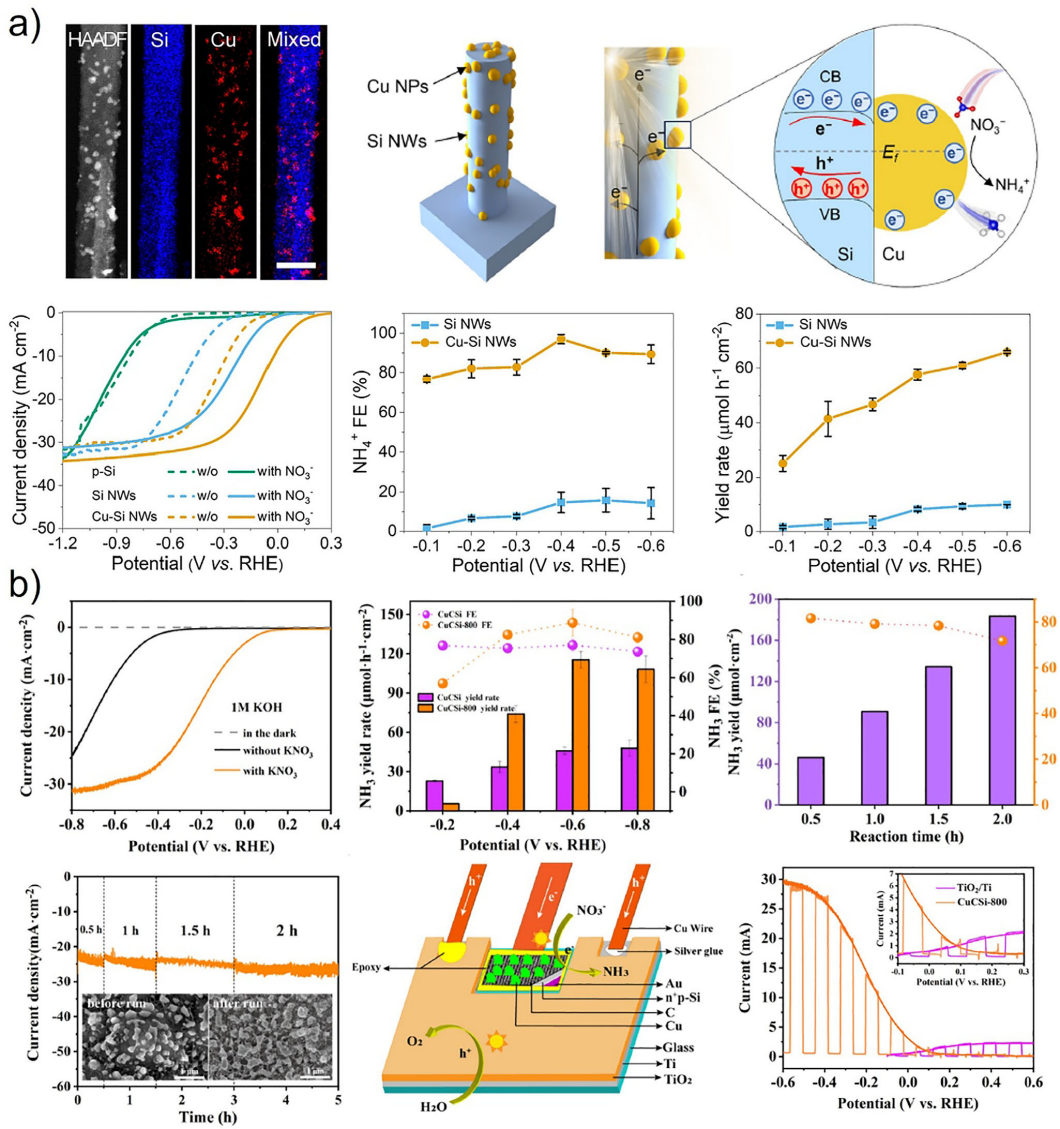
Selected b‐Si‐based photoelectrodes for PEC‐NO_3_RR. (a) Cu‐decorated Si NWs for acidic nitrate reduction. HAADF‐STEM and EDS mapping images confirm the uniform loading of Cu NPs on Si NWs, while the schematic illustrates interfacial electron transfer from Si to Cu active sites for NO_3_^−^‐to‐NH_4_^+^ conversion. LSV curves, NH_4_^+^ faradaic efficiencies and NH_4_^+^ yield rates compare p‐Si, Si NWs, and Cu–Si NWs in 0.5 m H_2_SO_4_ containing 100 mM NO_3_^−^ under simulated solar illumination. Reproduced with permission [50]. Copyright 2024, Royal Society of Chemistry. (b) Hierarchical Cu/C/b‐Si photocathode and CuCSi‐800|TiO_2_ tandem device for alkaline PEC‐NO_3_RR. J–V characteristics, potential‐dependent NH_3_ yield rates and faradaic efficiencies, time‐dependent NH_3_ production, chronoamperometric stability and photocurrent matching collectively demonstrate efficient solar‐driven ammonia synthesis from nitrate. Adapted with permission [125]. Copyright 2024, Elsevier B.V.

Recent studies have also begun to address PEC‐NO_3_RR at the full‐device level [125]. This direction is important because nitrate reduction at the photocathode must be coupled with a complementary anodic reaction to minimize or eliminate external bias. Ding et al. demonstrated this concept by constructing a bias‐free Cu/C/Si–TiO_2_ tandem system for solar‐to‐ammonia conversion. As shown in Figure 7b, the coplanar tandem device integrated a hierarchical Cu/C/Si photocathode with a TiO_2_ photoanode. The photocathode was prepared by introducing a carbon layer onto n^+^p‐Si and subsequently depositing Cu as the NO_3_RR cocatalyst. After thermal treatment at 800°C, the Cu layer was transformed into dispersed Cu NPs on the C/Si surface. The carbon interlayer improved electrical contact, stabilized the Si surface, and provided anchoring sites for Cu NPs. In the tandem configuration, the Cu/C/Si side drove nitrate reduction to NH_3_, while the TiO_2_ side supplied the complementary oxygen evolution.

The operation of this tandem system relied on both interfacial catalysis and photocurrent matching between the two photoelectrodes. Operando analyses suggested that photogenerated electrons accumulated at Cu sites and generated Lewis‐acidic Cu centers, which promoted NO_3_^−^ adsorption and subsequent hydrogenation toward ammonia. In a three‐electrode configuration, the optimized CuCSi‐800 photocathode delivered an NH_3_ yield rate of 115.3 µmol h^−1^ cm^−2^ with a faradaic efficiency of 88.8% at −0.6 V vs RHE in alkaline electrolyte. The reliability of NH_3_ production was further confirmed through dark and open‐circuit control experiments, together with ^15^NO_3_^−^ isotope‐labeling measurements. Consistent NH_3_ quantification obtained from the indophenol blue method and ^1^H NMR further supported that the detected ammonia originated from PEC nitrate reduction. When coupled with the TiO_2_ photoanode, the integrated tandem device achieved unbiased solar‐to‐ammonia conversion with an NH_3_ yield rate of 13.1 µmol h^−1 ^cm^−2^, a faradaic efficiency of 93.8% and a solar‐to‐ammonia efficiency of approximately 1.5%. This example shows that b‐Si‐based PEC‐NO_3_RR should be evaluated not only at the photocathode level but also within full‐cell architectures where charge balance and photocurrent matching become decisive.

Despite these advances, high catalytic activity and tandem‐device integration alone are insufficient for practical b‐Si‐based PEC‐NO_3_RR. Textured Si can provide strong light absorption and high photocurrent, but it remains vulnerable to corrosion and parasitic passivation in acidic or alkaline nitrate‐containing electrolytes. Zhou et al. addressed this issue by introducing an oxide/metal multilayer protection strategy for b‐Si photocathodes [126]. The central idea was to decouple the usual trade‐off between carrier transport and chemical durability. A very thin oxide layer can support efficient tunnelling but often lacks long‐term stability, whereas a thick oxide layer improves protection but blocks carrier transport.

The protected photocathode was constructed as C(TF)_6_CSi, consisting of an n^+^p‐Si light absorber, a carbon electron‐transfer layer, a six‐repeat TiO_2_/Fe protection layer and a FeCu alloy NO_3_RR cocatalyst. In this notation, TF represents one TiO_2_/Fe repeating unit. The repeated oxide/metal structure created multiple carrier‐tunnelling paths across the protection layer while maintaining sufficient total thickness for corrosion resistance. Thus, the multilayer did not function only as a passive barrier. It acted as an electronically engineered interlayer that regulated charge transport from b‐Si to the catalytic surface. In 1.0 m KOH containing 0.05 m KNO_3_, the C(TF)_6_CSi photocathode reached a saturation photocurrent density of −19.7 mA cm^−2^ and delivered an NH_3_ yield rate of 0.42 mg cm^−2^ h^−1^ with a faradaic efficiency of 77.3% at 0 V vs RHE. Electrochemical impedance and intensity‐modulated photocurrent analyses indicated lower charge‐transfer resistance and faster carrier transport compared with photocathodes containing too fewer or excessive oxide/metal repeats. The same architecture also improved durability, with an estimated lifetime exceeding 100 h under alkaline NO_3_RR conditions.

### 3.4 Nitrogen Reduction Reaction

Compared with nitrate reduction, PEC‐NRR offers a more direct but considerably more challenging route to solar‐driven ammonia synthesis because it uses molecular N_2_ as the nitrogen source. In this respect, PEC‐NRR shares the same feedstock as the Haber–Bosch process but aims to convert N_2_ into NH_3_ under ambient or mild conditions using photogenerated charge carriers. The main difficulty arises from the intrinsic inertness of N_2_, including its strong N≡N triple bond, nonpolar molecular structure and low solubility in aqueous electrolytes. In addition, the proton‐coupled multielectron reduction of N_2_ competes severely with HER, which is often more favorable on photocathode surfaces. Therefore, efficient PEC‐NRR requires catalytic interfaces that can activate N_2_, stabilize key intermediates such as N_2_, NNH, and *NH_x_, and suppress nonselective proton reduction [31, 127].

Within this demanding reaction context, b‐Si should be viewed not simply as a high‐surface‐area light absorber but as a platform for coupling photocarrier generation with N_2_‐activation chemistry. Accordingly, the interfacial design of b‐Si for NRR should prioritize efficient electron delivery to N_2_‐activation sites while minimizing surface recombination and competing proton reduction. Its effectiveness therefore depends on how the textured Si surface is integrated with catalysts or mediator‐forming interfaces that can extract photogenerated electrons and direct them toward N_2_ fixation. Without a suitable catalyst, b‐Si alone provides limited capability for molecular N_2_ activation because of the high kinetic barrier of N≡N bond cleavage and strong competition from HER. Its response therefore provides a necessary baseline for assessing the catalytic contribution of metal nanoparticles or mediator‐based interfaces. In addition, rigorous control experiments and isotope‐labeling measurements are particularly important in PEC‐NRR to verify that detected NH_3_ originates from N_2_ reduction rather than background nitrogen contamination. Recent b‐Si‐based PEC‐NRR studies can be broadly divided into two strategies. The first involves direct N_2_ activation on metal‐modified b‐Si photocathodes, where plasmonic or catalytic metal NPs are coupled to light‐trapping Si [128, 129]. The second uses mediated N_2_ fixation through alkali‐metal redox cycles, where photogenerated electrons drive Li^+^ or Na^+^ reduction before nitride formation and subsequent protonation [130, 131]. These two approaches highlight different roles of b‐Si in PEC‐NRR, ranging from direct semiconductor/cocatalyst interface engineering to photoassisted mediator cycling.

One of the earliest demonstrations of b‐Si‐based PEC‐NRR used a plasmon‐enhanced Au/b‐Si/Cr photoelectrode for N_2_‐to‐NH_3_ conversion [129]. In this configuration, p‐type Si was transformed into vertically aligned b‐Si NW arrays by dry etching, while Au NPs were deposited on the nanostructured surface as plasmonic and catalytic sites for N_2_ reduction. A Cr layer was placed on the backside as a hole sink and sacrificial anodic component. The b‐Si nanowire morphology strongly suppressed optical reflection and increased the surface area available for Au decoration. Compared with planar Si, the b‐Si electrode produced much higher ammonia yield, suggesting that morphology control is essential for improving solar‐driven N_2_ fixation. Au NPs further promoted charge separation and provided active sites for N_2_ reduction, while the backside Cr layer assisted hole extraction and charge balance across the PEC cell. The Au/b‐Si/Cr system produced NH_3_ at 13.3 mg m^−2^ h^−1^ under ambient pressure with 2 sun illumination, and the yield increased to 60 mg m^−2^ h^−1^ at 7 atm N_2_. This pressure‐dependent behavior indicates that N_2_ solubility and mass transport near the nanostructured interface are major limitations in aqueous PEC‐NRR. Although the efficiency remained far from practical ammonia production, this study established b‐Si as a viable light‐harvesting platform for solar‐driven N_2_ reduction.

Following this device‐level demonstration, Ag‐modified b‐Si photocathodes were developed to clarify the role of local metal/semiconductor interface engineering [128]. Vertically aligned b‐Si NW arrays were decorated with Ag NPs, producing a photocathode in which b‐Si served as a broadband absorber and large surface area scaffold, while Ag provided catalytic N_2_ reduction sites and plasmonic enhancement. Ag deposition time strongly affected the balance between catalytic site density and photon access to the Si absorber. Insufficient Ag loading limited N_2_ activation, whereas excessive Ag coverage partially screened the photoabsorber. Under optimized deposition conditions, well‐dispersed Ag NPs enabled catalytic contact without severe optical blocking. The Ag/b‐Si junction promoted interfacial charge separation by facilitating electron migration toward Ag NPs, where N_2_ reduction could proceed more favorably. This interpretation was supported by increased transient photocurrent response, reduced charge‐transfer resistance and prolonged carrier lifetime compared with bare b‐Si. The optimized photocathode achieved an NH_3_ yield rate of 2.87 µmol h^−1^ cm^−2^ at −0.2 V vs RHE and a maximum faradaic efficiency of 55.05% at −0.1 V vs RHE. At more negative potentials, NRR performance decreased because HER became increasingly competitive, showing that the Ag/b‐Si interface is effective only within an appropriate potential window. The reliability of NH_3_ detection was further examined through blank‐control measurements, in which the electrolyte after PEC‐NRR was compared with a blank sample using the indophenol method. These control measurements supported the assignment of the detected NH_3_ to the PEC‐NRR process rather than background contamination. The electrode also maintained stable photocurrent over 24 h with negligible morphological and chemical changes. These results show that b‐Si‐based PEC‐NRR requires not only strong light trapping but also careful control of cocatalyst loading, interfacial band bending and HER competition.

Although Au‐ and Ag‐modified b‐Si photocathodes demonstrate direct PEC‐NRR, the kinetic barrier for N_2_ activation remains high. Metal‐mediated PEC‐NRR has therefore emerged as an alternative strategy. In this pathway, N_2_ is not reduced only through direct adsorption and hydrogenation on the photocathode surface. Instead, photogenerated electrons first reduce an alkali‐metal cation to a metallic mediator, which then reacts with N_2_ to form a nitride intermediate. Subsequent protonation produces NH_3_ and regenerates the metal cation for the next cycle. This mediated pathway can lower the kinetic barrier for N_2_ activation and shift the reaction away from conventional surface‐bound N_2_ hydrogenation.

As shown in Figure 8a, Li‐mediated PEC‐NRR was demonstrated using a hierarchical PdCu/TiO_2_/Si photocathode [130]. In this architecture, nanostructured n^+^p‐Si served as the light absorber, TiO_2_ acted as a protective and interfacial layer, and PdCu alloy functioned as the cocatalyst for Li^+^ adsorption and reduction. The TiO_2_ layer was not primarily introduced to enhance light absorption. Instead, it improved interfacial stability and provided an adhesion layer for the PdCu cocatalyst. The porous PdCu layer supplied active sites for Li^+^ reduction while still allowing light penetration into the Si absorber. Under illumination, electrons generated in Si were transferred through TiO_2_ to the PdCu surface, where Li^+^ reduction and subsequent Li‐mediated N_2_ fixation occurred.

**FIGURE 8:**
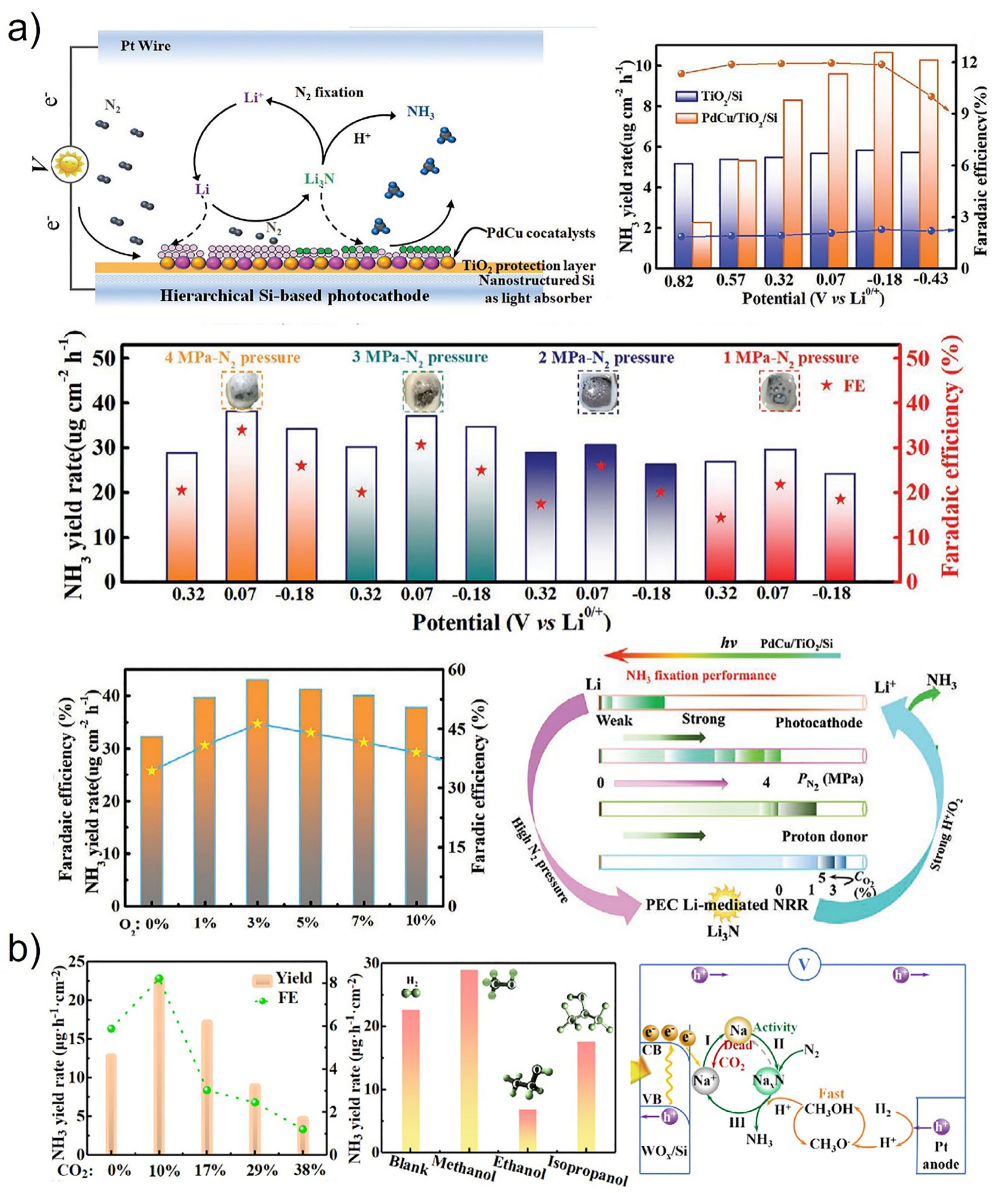
Representative examples of b‐Si‐based PEC‐NRR. (a) Schematic illustration and performance overview of Li‐mediated PEC‐NRR on a hierarchical PdCu/TiO_2_/Si photocathode. The figure presents the Li‐mediated N_2_‐to‐NH_3_ conversion pathway, potential‐dependent NH_3_ yield rate and faradaic efficiencies for PdCu/TiO_2_/Si and TiO_2_/Si photocathodes, N_2_ pressure‐dependent performance of PdCu/TiO_2_/Si, and the effect of O_2_ concentration in mixed N_2_/O_2_ gas under 1 sun irradiation at 0.07 V versus Li^0/+^. The enhanced performance is attributed to optimization of the Li cycle, involving Li deposition, Li_3_N formation, Li_3_N decomposition, and NH_3_ release. Adapted with permission [130]. Copyright 2023, Wiley‐VCH GmbH. (b) Effect of gas composition and proton shuttles on Na‐mediated PEC‐NRR. The figure summarizes the CO_2_ concentration‐dependent NRR performance in mixed gas at 0.8 V vs Na^+^/Na and the proton shuttle‐dependent NH_3_ yield rate under 1 sun illumination, 1 MPa mixed gas of 87% N_2_, 3% H_2_, and 10% CO_2_, and 0.8 V vs Na^+^/Na. The insets show the molecular structures of H_2_, MeOH, EtOH, and IPA. The schematic illustration depicts the enhanced Na‐mediated PEC‐NRR mechanism achieved by regulating the Na cycle, including active Na formation, dead Na suppression, intermediate hydrogenation and Na^+^ regeneration. Adapted with permission [131]. Copyright 2025, Elsevier B.V.

The performance trends in Figure 8a show that Li‐mediated PEC‐NRR depends strongly on potential, N_2_ pressure and Li‐cycle regulation. Under ambient N_2_, the PdCu/TiO_2_/Si photocathode showed higher NH_3_ yield rate and faradaic efficiency than TiO_2_/Si, confirming the role of PdCu in promoting Li^+^ reduction. However, excessively negative potentials decreased performance because uncontrolled Li deposition and thick Li plating could block light absorption and hinder productive Li cycling. Increasing N_2_ pressure improved NH_3_ formation by increasing N_2_ availability and facilitating Li_3_N formation. At 3 MPa N_2_, the NH_3_ yield rate reached 36.98 µg cm^−2 ^h^−1^ with a faradaic efficiency of 30.66% at 0.07 V vs Li^0/+^. Further improvement was achieved by regulating Li_3_N conversion rather than simply increasing Li deposition. A stronger proton donor such as trifluoroethanol accelerated Li_3_N protonation by promoting Li─N bond cleavage and N─H bond formation. Introducing a small amount of O_2_ also promoted Li_3_N decomposition and refreshed the Li cycle. Under optimized mixed gas conditions with 0.12 MPa O_2_ and 3.88 MPa N_2_, the system achieved an NH_3_ yield rate of 43.09 µg cm^−2^ h^−1^ and a faradaic efficiency of 46.15% at 0.07 V vs Li^0/+^. The origin of the produced NH_3_ was rigorously verified through open‐circuit, photocathode‐free, dark, and Ar‐control experiments, in which no detectable NH_3_ formation was observed. In addition, ^15^N_2_ isotope‐labeling combined with NMR analysis confirmed that the nitrogen in the generated ammonia originated from the supplied N_2_. This study shifted b‐Si‐based PEC‐NRR from direct surface catalytic reduction toward mediator‐controlled reaction cycling.

As a further extension of metal‐mediated PEC‐NRR, Na‐mediated PEC‐NRR has been explored to address some limitations of Li‐based systems [131]. Li is costly, less abundant and associated with safety concerns, while Na is more earth‐abundant and has a less negative Na^+^/Na redox potential. In a Na‐mediated pathway, photoexcited electrons reduce Na^+^ to metallic Na. The metallic Na then reacts with N_2_ to form sodium nitride intermediates, which are subsequently hydrogenated to produce NH_3_ while regenerating Na^+^. The corresponding PEC system used a hierarchical WO_x_/Si photocathode in NaClO_4_‐propylene carbonate electrolyte. The Si substrate was converted into an n^+^p‐Si photocathode with a nano‐pyramidal surface through alkaline etching and phosphorus doping. This textured Si architecture provided light absorption and built‐in electric field formation. A WO_x_ layer was then introduced as a cocatalytic layer for Na^+^ adsorption and reduction. The coexistence of W^6+^/W^5+^ redox states in oxygen‐deficient WO_x_ facilitated Na^+^ adsorption and promoted metallic Na formation on the photocathode surface.

The main limitation of Na‐mediated PEC‐NRR is the low reversibility of Na deposition and stripping. During operation, part of the deposited Na can become inactive and accumulate as “dead” Na, which blocks fresh metallic Na sites and suppresses N_2_ activation. In addition, the sodium nitride intermediate, denoted as Na_x_N, is highly unstable and can decompose back to inactive Na before hydrogenation. To address these issues, CO_2_ was introduced into the mixed reaction gas, as summarized in Figure 8b. At an optimized CO_2_ concentration of 10%, CO_2_ helped disrupt the passivating dead‐Na layer and exposed fresh Na sites for N_2_ activation. Excessive CO_2_, however, decreased performance because stable Na_2_CO_3_ formation reduced the number of active metallic Na sites.

Figure 8b also shows that proton‐shuttle selection strongly influences Na‐mediated PEC‐NRR. Among the examined proton shuttles, methanol produced the highest NH_3_ yield rate because it accelerated Na_x_N hydrogenation by promoting Na─N bond cleavage and N─H bond formation. Ethanol and isopropanol led to lower activity, indicating that proton transfer kinetics is a key factor in the Na‐mediated pathway. With CO_2_‐assisted dead‐Na removal and methanol‐assisted Na_x_N hydrogenation, the WO_x_/Si photocathode achieved an NH_3_ yield rate of 29.0 µg h^−1 ^cm^−2^ at 0.8 V vs Na^+^/Na. Although this value remained lower than the best Li‐mediated PEC‐NRR performance, the study expanded mediated PEC‐NRR from Li chemistry to a more earth‐abundant Na‐based cycle. It also showed that efficient b‐Si‐related PEC‐NRR requires integrated control of photocathode design, mediator deposition, intermediate hydrogenation, and mediator regeneration. For PEC‐NO_3_RR and PEC‐NRR, the reported production rates, Faradaic efficiencies, and corresponding electrochemical conditions are compared in Table 4.

**TABLE 4:** Representative b‐Si‐based photoelectrodes for PEC‐NO_3_RR and PEC‐NRR.

Reaction	Device structure	Electrolyte	Key performance	Selectivity	Production (mmol m^−2^ h^−1^)	FE (%)	Stability	Ref.
NO_3_RR	O_SiNW/Au	0.5 m K_2_SO_4_ + 10 mm KNO_3_	J ≈ 0.3–0.5 mA cm^−2^ at 0.1 V vs RHE, V_onset_ ≈ 0.3 V vs RHE	NH_3_	≈ 4.1 at 0.1 V vs RHE	95.6 at 0.2 V vs RHE	—	[61]
	Cu–Si NWs	0.5 m H_2_SO_4_ + 0.1 m NO_3_^−^	J = 31.06 mA cm^−2^ at 0.4 V vs RHE, J_sat_ = 34.29, V_onset_ = 0.3 V vs RHE	NH_4_^+^	659.1 at −0.6 V vs RHE	97.03 at −0.4 V vs RHE	> 36 h	[50]
	CuCSi‐800 (Cu NPs/C/n^+^p‐Si‐800)	1 m KOH + 50 mm KNO_3_	J ≈ 20–25 mA cm^−2^ at −0.6 V vs RHE, V_onset_ ≈ 0.2 V vs RHE	NH_3_	1153 at −0.6 V vs RHE	88.8 at −0.6 V vs RHE	2 h	[125]
	CuCSi‐800|TiO_2_	1 m KOH + 50 mm KNO_3_	J > 2 mA cm^−2^ at 0 V, V_onset_ = 0 V, bias‐free	NH_3_	131 at 0 V cell bias (bias‐free)	93.8 at 0 V cell bias (bias‐free)	10 min	[125]
	FeCu/(TF)_6_/C/n^+^p‐Si	1 m KOH + 50 mm KNO_3_	J_sat_ = 19.7 mA cm^−2^, J = 10 mA cm^−2^ at 0.055 V vs RHE, V_onset_ ≈ 0.45 V vs RHE	NH_3_	246.6 at 0 V vs RHE	77.3 at 0 V vs RHE	12 h	[126]
NRR	Au/b‐Si NW/Cr	150 ppm Na_2_SO_3_	—	NH_3_/NH_4_^+^	0.781 without applied potential; 2 sun	—	18 h	[129]
	Ag NPs/b‐Si NW	0.1 m Na_2_SO_4_	J ≈ 0.20 mA cm^−2^ at −0.2 V vs RHE; V_onset_ ≈ 0.3 V vs RHE	NH_3_	28.7 at −0.2 V vs RHE	55.05 at −0.1 V vs RHE	24 h	[128]
	PdCu/TiO_2_/n^+^p‐Si	1 m LiClO_4_‐PC	0.07 V vs Li^0/+^	NH_3_	25.3 at 0.07 V vs Li^0^/^+^	46.15 at 0.07 V vs Li^0^/^+^	20 h	[130]
	WO_x_/b‐Si	0.6 m NaClO_4_‐PC	0.8 V vs Na^+^/Na	NH_3_	17.0 at 0.8 V vs Na^+^/Na	—	—	[131]

### 3.5 CO_2_ Reduction Reaction

The increasing demand for carbon‐neutral energy systems has stimulated extensive interest in solar‐driven CO_2_ conversion. PEC‐CO_2_RR is a promising strategy for transforming CO_2_ into carbon‐based fuels and chemical feedstocks by combining semiconductor light absorption with electrocatalytic CO_2_ reduction [32, 132]. Unlike HER, CO_2_RR involves multiple proton‐coupled electron transfer pathways and can produce a wide range of products. The reaction generally proceeds through CO_2_ adsorption, interfacial charge transfer and the formation of key intermediates such as *CO_2_^−^, *COOH, *OCHO, *CO, *CHO, and *OCCO. Depending on the catalyst surface and local reaction environment, CO_2_ can be converted into C1 products such as CO, formate (HCOO^−^), methane (CH_4_) and methanol (CH_3_OH), or C2 products such as ethylene (C_2_H_4_) and ethanol (C_2_H_5_OH). However, selective CO_2_ conversion remains difficult because CO_2_ is thermodynamically stable and kinetically inert. In aqueous electrolytes, HER also competes strongly with CO_2_RR on photocathode surfaces [133, 134].

For b‐Si‐based PEC‐CO_2_RR, the central design issue is not simply to enhance light absorption but to deliver photogenerated electrons selectively to CO_2_ reduction pathways. Without a catalyst, b‐Si generally exhibits limited intrinsic selectivity toward CO_2_ reduction and can preferentially drive the competing HER in aqueous electrolytes. This behavior provides an important baseline for evaluating how cocatalysts, molecular modifiers, and interfacial layers redirect photogenerated electrons toward specific CO_2_RR pathways. The textured b‐Si surface can facilitate catalyst integration, enlarge the semiconductor/catalyst interface and support efficient charge delivery to catalytic sites. At the same time, its enlarged surface area can intensify surface recombination, chemical instability and parasitic HER if the interface is not carefully controlled. Therefore, b‐Si‐based PEC‐CO_2_RR systems require rational integration of passivation layers, molecular modifiers and CO_2_RR‐selective cocatalysts [135]. Recent studies have mainly focused on regulating the local reaction microenvironment, improving semiconductor/catalyst contact and tuning cocatalyst composition to direct product selectivity toward CO, formate and more deeply reduced hydrocarbon products.

One strategy to improve CO selectivity is to regulate the molecular environment around metal cocatalysts. Kan et al. demonstrated this concept using 2‐aminobenzenethiol‐functionalized Ag‐decorated b‐Si photocathodes [136]. Nanoporous p‐type b‐Si was prepared by Ag‐assisted electroless etching and then decorated with Ag NPs. In this system, b‐Si enhanced light absorption and provided a large surface area for Ag loading, while Ag served as a CO‐selective cocatalyst by stabilizing the *COOH intermediate. However, the enlarged interface of b‐Si also increased the number of sites where HER could occur. To suppress this competing pathway and improve CO_2_ activation, a molecularly thin 2‐aminobenzenethiol (2‐ABT) layer was adsorbed onto the Ag surface. The thiol group anchored the molecule to Ag through Ag–S bonding, while the ortho‐positioned amino group was located close to the catalytic surface. This configuration was proposed to assist CO_2_ binding and proton transfer near Ag sites.

The 2‐ABT‐modified b‐Si/Ag photocathode exhibited an onset potential of approximately −50 mV vs RHE for CO_2_ reduction under illumination, corresponding to an anodic shift of about 150 mV compared with the unmodified electrode. The FE_CO_ increased from approximately 50% for b‐Si/Ag to about 75% after 2‐ABT functionalization. The CO_2_ reduction current also increased, indicating that the molecular layer accelerated interfacial CO_2_RR kinetics rather than merely blocking HER. Control experiments with other aromatic thiols and amines showed weaker effects, confirming that the local molecular geometry near the Ag surface was important for promoting CO formation. This result shows that the selectivity of b‐Si‐based PEC‐CO_2_RR can be improved by molecular‐level regulation of the catalyst/electrolyte interface.

In addition to controlling the local reaction environment, understanding how photogenerated carriers are energetically delivered to catalytic sites is essential for evaluating b‐Si‐based PEC‐CO_2_RR photocathodes. Suo et al. addressed this issue using a metal–insulator–semiconductor (MIS) photocathode composed of p‐type b‐Si, a native SiO_x_ layer and Ag NPs [137]. In this b‐p‐Si/SiO_x_/Ag structure, b‐Si functioned as the light absorber, SiO_x_ served as an interfacial tunnelling layer and Ag NPs acted as CO_2_RR catalytic sites. The key contribution of this study was the direct measurement of the quasi‐Fermi level of Ag under operating conditions. By selectively adsorbing 4‐mercaptobenzonitrile on Ag NPs and monitoring its vibrational Stark shift with surface‐enhanced Raman spectroscopy, the photovoltage at the Si/SiO_x_/Ag junction was estimated to be approximately 0.59 V. This value represents the energetic driving force available at the metal catalytic sites rather than an average photovoltage over the whole electrode. The result highlights the importance of site‐selective characterization for heterogeneous b‐Si photocathodes, where catalytically active and inactive surface regions can coexist.

The importance of semiconductor/cocatalyst contact was further demonstrated using Au‐modified b‐Si photocathodes for selective CO production [138]. Au NPs were incorporated onto textured p‐type Si as CO‐selective catalytic sites. Unlike continuous Au films that can block incident light, isolated Au NPs preserve photon access to the Si absorber while providing CO_2_RR active sites. The Au NPs contained controllable Au(111)/Au(200) boundaries, and density functional theory calculations suggested that these boundaries lower the energy barrier for *COOH formation. Thus, the enhanced CO_2_‐to‐CO activity originated from both the intrinsic catalytic property of Au and the engineered facet boundary structure.

Further improvement was achieved by partially embedding the Au NPs into the b‐Si surface through an additional etching step. This buried Au/b‐Si configuration strengthened the physical and electronic contact between Au and Si. As a result, the charge transfer resistance decreased from 117.4 Ω for Au1/Si to 64.2 Ω for b‐Au1/Si, indicating faster interfacial electron transfer. The embedded structure also suppressed interfacial recombination, as reflected by reduced transient photocurrent spikes under chopped illumination. In PEC‐CO_2_RR, the optimized b‐Au1/Si photocathode achieved a photocurrent density of −13.1 mA cm^−2^ at −1.0 V vs RHE, an FE_CO_ of 82.2% and operational stability for more than one week. During long‐term operation, the FE_CO_ remained above 76.1% after 120 h. These results emphasize that intimate semiconductor/cocatalyst contact is critical for reducing charge‐transfer resistance and maintaining selective CO production on b‐Si photocathodes.

Beyond Ag‐ and Au‐based CO‐selective photocathodes, cocatalyst composition has been tuned to redirect CO_2_RR toward formate production. A representative example is the Bi–Sn/SiNW photocathode, in which p‐type Si nanowire arrays were integrated with a bimetallic cocatalyst [139]. The Si NW array was prepared by MACE to reduce optical reflection and provide a high‐area framework for cocatalyst loading. Bi NPs were first deposited on the SiNW surface, followed by Sn deposition to form a core–shell‐like Bi–Sn cocatalyst structure. Bi served as the main formate‐selective component by favoring the *OCHO‐mediated pathway, while the Sn layer modified the surface environment and reduced direct contact between Si and water. This configuration helped suppress HER and promoted formate formation.

The Bi–Sn/SiNW photocathode showed more positive onset behavior and higher photocurrent density than SiNW, Bi/SiNW, and Sn/SiNW photocathodes. It achieved a formate faradaic efficiency of 88.67% and a formate production rate of 80.07 µmol h^−1^ cm^−2^ at −1.02 V vs RHE. At more negative potentials, the formate production rate increased but the faradaic efficiency decreased because excess electrons were increasingly consumed by HER. Compared with Ag‐ and Au‐modified photocathodes that primarily favor the COOH‐mediated CO pathway, the Bi–Sn/SiNW system shifted the dominant product toward formate by stabilizing OCHO intermediates. This transition from noble‐metal CO production to non‐noble bimetallic formate production shows that product selectivity in b‐Si‐based PEC‐CO_2_RR can be redirected through cocatalyst composition and intermediate‐binding control.

More demanding CO_2_RR pathways require deeper multielectron reduction beyond CO or formate. In this context, textured Si photocathodes have been engineered with tandem catalyst structures to promote methane formation. Wu et al. developed a pyramid‐textured b‐Si photocathode coupled with Cu/Ag–Cu bilayer catalysts for selective PEC CO_2_‐to‐CH_4_ conversion [140]. As shown in Figure 9a, bare pyramid b‐Si mainly produced H_2_, indicating that the Si surface alone could not efficiently direct photogenerated electrons toward CO_2_ reduction. Ag modification promoted CO formation, while Cu‐containing catalysts enabled further proton‐coupled reduction. However, single‐metal or simply mixed catalyst layers still showed incomplete control over product distribution. Therefore, the central design concept was to spatially separate CO_2_ activation, CO formation, CO hydrogenation and *CHO. The CuPD/Ag–CuCD|b‐Si photocathode contained a bottom Ag–Cu layer introduced by chemical deposition and an upper Cu layer added by photoelectrodeposition. The bottom Ag–Cu layer promoted CO_2_ activation and CO formation, whereas the upper Cu layer facilitated further hydrogenation of CO to CHO, a key intermediate for CH_4_ formation. The free‐energy diagram and in situ infrared analysis in Figure 9a support this tandem pathway by identifying intermediates such as *CO, *COOH, *CHO, and bicarbonate‐related species under applied bias. The operando synchrotron‐radiation FTIR configuration and mechanistic model in Figure 9b further connect the bilayer catalyst structure with the CO_2_‐to‐CH_4_ reaction pathway. At −1.1 V vs RHE, the CuPD/Ag–CuCD|b‐Si photocathode reached a working current density of approximately −32.9 mA cm^−2^ and a CH_4_ selectivity of about 60%. The ^13^CO_2_ isotope‐labeling combined with GC–MS analysis further confirmed that the detected CH_4_ and CO originated from CO_2_ reduction. This result indicates that high CH_4_ selectivity requires spatially organized catalytic functions rather than the simple coexistence of Ag and Cu components.

**FIGURE 9:**
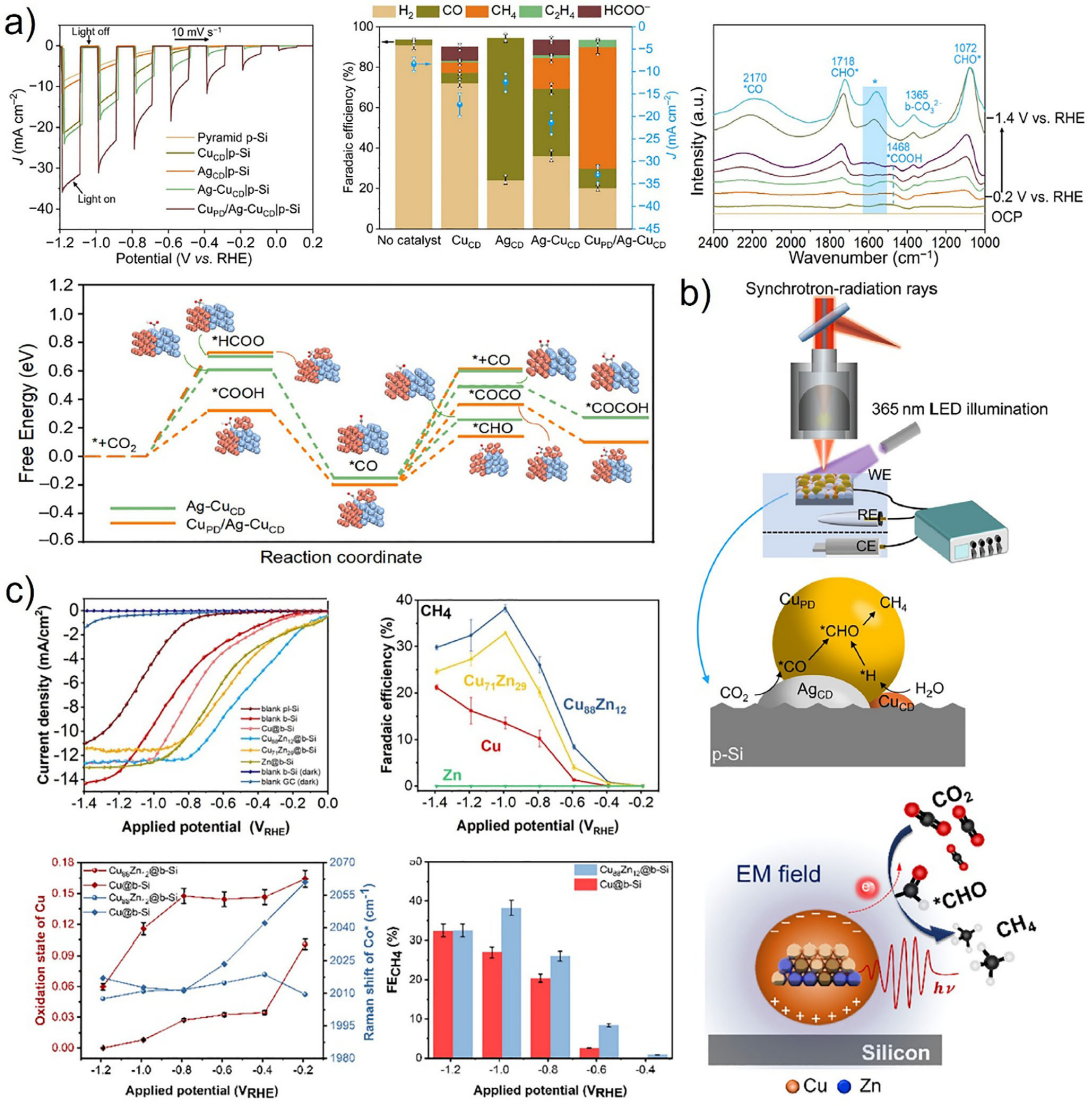
Representative b‐Si‐based photocathodes for PEC‐CO_2_RR. (a) Performance and mechanistic analysis of a pyramid‐textured CuPD/Ag–CuCD|b‐Si photocathode for CO_2_‐to‐CH_4_ conversion. J–V curves, product distributions, operando synchrotron‐radiation FTIR spectra and DFT calculations show that the Ag–Cu/Cu bilayer tandem catalyst promotes CO_2_ activation, *CO formation and subsequent *CO hydrogenation toward CH_4_. (b) Operando FTIR configuration and proposed tandem reaction pathway over the CuPD/Ag–CuCD|Si photocathode. Adapted with permission [140]. Copyright 2026, Wiley‐VCH GmbH. (c) PEC‐CO_2_RR performance and mechanism of CuZn alloy‐modified b‐Si photocathodes. LSV curves, CH_4_ faradaic efficiencies, Cu oxidation‐state analysis and Raman shifts of *CO indicate that CuZn alloying stabilizes Cu^0^, modulates *CO binding and promotes *CHO‐mediated CH_4_ formation under illumination. Adapted with permission [141]. Copyright 2026, Elsevier B.V.

A more direct b‐Si‐based approach to methane‐rich PEC‐CO_2_RR was demonstrated using CuZn alloy‐modified b‐Si photocathodes [141]. In this system, hierarchical porous b‐Si acted as the photoabsorbing scaffold, while CuZn alloy nanoparticles served as CO_2_RR catalytic sites. As shown in Figure 9c, planar Si, blank b‐Si, Zn@b‐Si, Cu@b‐Si, Cu_88_Zn_12_@b‐Si, and Cu_71_Zn_29_@b‐Si photocathodes were compared in CO_2_‐saturated KHCO_3_ electrolyte. Unmodified planar Si and blank b‐Si exhibited limited cathodic photocurrent, whereas metal‐modified b‐Si photocathodes showed enhanced PEC responses. This blank b‐Si response provides a catalyst‐free baseline for assessing the contribution of Cu–Zn alloying to CO_2_RR activity and product selectivity. Among them, Cu_88_Zn_12_@b‐Si showed the most favorable methane‐forming behavior. Compared with Cu@b‐Si, Cu_88_Zn_12_@b‐Si lowered the onset potential for CH_4_ formation by approximately 0.2 V and increased the CH_4_ faradaic efficiency by 2.28 times at −0.99 V vs RHE. The optimized Cu_88_Zn_12_@b‐Si photocathode reached a maximum CH_4_ faradaic efficiency of approximately 38%–40%. The carbon origin of the products was further verified by a ^13^C isotope‐tracer experiment combined with GC–MS analysis, confirming that the detected carbon products originated from CO_2_ reduction.

The product distribution depended strongly on the Cu/Zn composition and applied potential. Zn@b‐Si mainly favored less reduced C1 products such as CO and formate, whereas Cu‐containing b‐Si photocathodes enabled hydrocarbon formation. Excessive Zn incorporation in Cu_71_Zn_29_@b‐Si decreased methane selectivity, indicating that an appropriate Cu/Zn ratio is necessary to balance CO_2_ activation, *CO retention and subsequent hydrogenation. Mechanistic analyses in Figure 9c showed that Cu_88_Zn_12_@b‐Si stabilized metallic Cu^0^ more effectively than Cu@b‐Si under PEC‐CO_2_RR conditions. Raman shifts of adsorbed *CO further confirmed that Zn incorporation modified the *CO binding environment on Cu sites. This optimized *CO adsorption promoted further proton‐coupled reduction rather than CO desorption, thereby facilitating *CHO formation and directing the pathway toward CH_4_‐rich products. The performance of representative b‐Si‐based photocathodes for PEC‐CO_2_RR is summarized in Table 5 with emphasis on product formation and selectivity.

**TABLE 5:** Representative b‐Si‐based photoelectrodes for PEC‐CO_2_RR.

Device structure	Electrolyte	Key performance	Selectivity	Production (mmol m^−2^ h^−1^)	FE (%)	Stability	Ref.
b‐Si/Ag/2‐ABT	CO_2_‐sat. 0.2 m KHCO_3_	J ≈ 15.5 mA cm^−2^, J_CO_ = 11.7 mA cm^−2^, V_onset_ ≈ −0.05 V vs RHE	CO	≈ 2180	≈ 75	−	[136]
Au_3_Cu NP/TiO_2_/n^+^p‐SiNW	CO_2_‐sat. 0.1 m KHCO3	J_CO_ = 2.2–3.8 mA cm^−2^ at −0.20 to −0.37 V vs RHE	CO	≈ 410–709	≈ 80	18 h	[142]
b‐Au1/Si	CO_2_‐sat. 0.1 m KHCO_3_	J = −13.1 mA cm^−2^ at −1.0 V vs RHE, J_CO_ = −4.1 mA cm^−2^ at −0.4 V vs RHE, V_onset_ = 0.22 V vs RHE	CO	≈ 765	82.2	> 1 week	[138]
Cu NP/n^+^p‐SiNW	0.1 m KHCO3	J_C2H4_ > 2.5 mA cm^−2^ at −0.50 V vs RHE, V_ph_ ≈ 350 mV	C_2_H_4_	> 78	≈ 25	50 h	[143]
Bi–Sn/SiNW	CO_2_‐sat. 0.1 m KHCO_3_	J ≈ −18 mA cm^−2^ at −1.5 V vs RHE, V_onset_ = −0.10 V vs RHE	HCOO^−^	800.7	88.67	2 h	[139]
Ni@In/p‐SiNW	0.1 m KHCO_3_	J_HCOO_ ≈ 3.1 mA cm^−2^ at −1.2 V vs RHE	HCOO^−^	580	87	−	[144]
Sn/p‐SiNW	0.1 m KHCO_3_	J_HCOO_ ≈ 1.0 mA cm^−2^ at −0.875 V vs RHE	HCOO^−^	189	88	3h	[62]
Cu_88_Zn_12_@b‐Si	CO_2_‐sat. 0.1 m KHCO_3_	J ≈ −12.5 mA cm^−2^ at −0.99 V vs RHE, J_sat_ ≈ −12.9 mA cm^−2^, V_CH4, onset_ ≈ −0.79 V vs RHE	CH_4_	≈ 233	40	10 h	[141]
CuPD/Ag–CuCD|b‐Si	CO_2_‐sat. 0.5 m KHCO_3_	J = −32.9 ± 1.9 mA cm^−2^ at −1.1 V vs RHE, V_onset_ = 0.16 V vs RHE	CH_4_	≈ 920	60.2 ± 3.4	10 h	[140]

## 4 Remaining Challenges of b‐Si in PEC Applications

Despite substantial progress in b‐Si‐based PEC systems, several challenges must still be addressed before b‐Si can serve as a reliable platform for solar fuel generation. Its ultralow reflectance, broadband light trapping and enlarged interfacial area are valuable for PEC operation. However, these structural advantages do not by themselves ensure high activity, selectivity or durability. Each target reaction, including HER, OER, NO_3_RR, NRR and CO_2_RR, imposes different requirements for band alignment, charge extraction, reaction kinetics and product control. The local reaction environment also changes with reactant concentration, gas evolution, bubble formation, and product transport [20, 21, 22]. Therefore, the remaining challenges should be considered as interconnected issues spanning chemical and electrochemical stability, the interplay between catalyst integration and light trapping, mass transport, reaction selectivity, and system‐level photovoltage constraints.

A central challenge is the trade‐off between optical enhancement and carrier recombination. Textured b‐Si can suppress reflection and increase photon absorption, yet the same micro‐/nanostructured surface can introduce abundant dangling bonds, surface states and defect‐rich regions. These sites promote carrier recombination and lower the fraction of photogenerated charges that reach the catalytic interface. This limitation becomes more pronounced in high aspect ratio structures because the enlarged surface area increases both electrolyte contact and defect‐mediated charge loss. Thus, the best PEC morphology is not necessarily the one with the lowest reflectance. It should instead balance light trapping with minority‐carrier lifetime, charge collection and interfacial reaction kinetics. This trade‐off relationship was discussed by the TiO_2_‐passivated b‐Si photoanode, in which the nanostructure was designed to maintain strong light absorption while limiting recombination losses. The result highlights that optical optimization should be evaluated together with carrier lifetime and interfacial charge collection rather than reflectance alone [19]. Future studies should correlate morphology‐dependent optical gains with recombination dynamics and reaction rates under realistic operating conditions. Beyond these morphology‐related trade‐offs, the chemical and electrochemical stability of b‐Si under practical PEC operating conditions represents another fundamental challenge.

### 4.1 Chemical and Electrochemical Instability in Harsh Environments

Si is chemically and electrochemically unstable in many aqueous electrolytes, a problem that becomes more pronounced in b‐Si because nanoscale curvature and large surface area make complete protection difficult. Oxide layers such as TiO_2_, HfO_2_ and NiO_x_ have been used to suppress corrosion and reduce surface recombination [19, 101, 102, 103, 125, 126]. However, protective layers can increase interfacial resistance, hinder charge transport or block active sites when they are too thick, poorly conductive or nonuniform. A representative example is the multilayer‐protected photocathode, in which an ultrathin oxide favored carrier tunneling but provided insufficient durability, whereas excessive protection thickness hindered charge transport. This case demonstrates that protection‐layer design in textured Si is fundamentally governed by a trade‐off between chemical stability and electronic transport [126]. In complex textured b‐Si, incomplete coating can leave exposed Si regions that act as local degradation sites. An effective protection layer should therefore be conformal, chemically robust, optically transparent, and electronically selective [21]. More importantly, the protection strategy should not be optimized solely for chemical robustness, because excessive protection can compromise charge extraction and catalytic accessibility. Thus, a key design challenge is to achieve sufficient interfacial protection while preserving efficient carrier transport across the entire complicated b‐Si architecture. Achieving these requirements in a scalable manner remains technically demanding. Long‐term stability remains a decisive barrier for practical PEC operation. Many b‐Si photoelectrodes show high initial photocurrent or Faradaic efficiency, but reported durability is often limited to tens or hundreds of hours. Practical systems require stable operation under fluctuating illumination, gas evolution, variable temperature, and aggressive electrolyte conditions [33]. Degradation may arise from Si oxidation, catalyst detachment, passivation layer failure, photocorrosion or interfacial reconstruction. In some adaptive junctions, controlled reconstruction can improve activity. In most cases, however, uncontrolled structural evolution causes performance decay. From a practical perspective, stability should therefore be evaluated not only by the duration of continuous operation but also by the ability of the protection and catalytic interfaces to maintain their structural and electronic functions under realistic operating fluctuations. Durable b‐Si photoelectrodes require protection schemes that preserve optical, electrical, and catalytic functions without introducing excessive resistance or fabrication complexity during prolonged operation.

### 4.2 Trade‐Off Between Catalyst Integration and Light Trapping

Catalyst integration presents another key challenge. The trade‐off between catalyst loading and light absorption is not unique to b‐Si and is generally encountered in PEC photoelectrodes, where increased catalyst coverage can improve reaction kinetics but excessive loading may introduce parasitic light absorption or optical shielding. In b‐Si, however, this issue becomes particularly important because its PEC advantage relies strongly on the preservation of a micro‐/nanostructured light‐trapping geometry. The large surface area of b‐Si is beneficial for catalyst loading, but excessive or poorly distributed catalyst coverage can screen incident light and reduce photon absorption [112, 115]. This limitation was observed in the Fe‐modified b‐Si photoanode, where increased catalyst coverage improved durability but reduced photocurrent through optical blocking [112]. A similar behavior was reported for Ag‐decorated b‐Si in NRR, where insufficient Ag loading limited catalytic site density, whereas excessive coverage screened the b‐Si absorber [128]. These examples indicate that catalyst coverage should be optimized spatially rather than simply maximized. Excessive or poorly controlled catalyst loading can also introduce parasitic recombination pathways, further emphasizing that catalyst coverage should be optimized rather than maximized. In highly textured b‐Si photoelectrodes, catalyst location across the nanostructured surface can influence local light absorption, charge‐carrier extraction, and recombination because the optical advantage of b‐Si depends strongly on preserving its light‐trapping geometry. Nanoparticle catalysts, bilayer structures and molecularly modified interfaces have been explored to address this balance, while general design rules remain insufficient. More systematic studies are needed to clarify how catalyst size, spatial distribution, penetration depth and semiconductor contact affect local band bending, charge transfer and reaction selectivity on textured Si surfaces [138, 140]. From a design perspective, catalyst amount, size, and spatial distribution should therefore be optimized together with the underlying light‐trapping architecture.

### 4.3 Mass Transport Limitations and Bubble Management

Mass transport is another underexplored issue. High aspect ratio b‐Si can improve electrolyte access to active sites, but narrow pores, dense nanowires and spike‐like textures can also impede reactant diffusion and product removal. Therefore, maximizing the geometric surface area of b‐Si does not necessarily maximize the electrochemically accessible area under operating conditions, particularly when diffusion through confined nanostructures becomes rate‐limiting. In gas‐involving reactions such as HER, NRR, and CO_2_RR, bubbles can block catalytic sites, scatter incident light and alter the local electrolyte composition. Bubble accumulation can be particularly detrimental in densely textured b‐Si because trapped gas pockets can reduce contact between electrode and electrolyte and locally disturb both optical and electrochemical conditions. Thus, bubble nucleation, growth and detachment should be considered as part of the electrode design rather than treated solely as a consequence of gas evolution [33]. For NO_3_RR and CO_2_RR, local reactant depletion or intermediate accumulation can further change activity and selectivity. The Au/b‐Si/Cr NRR system provides a clear example of this limitation, as the NH_3_ yield increased from 13.3 mg m^−2^ h^−1^ under ambient pressure to 60 mg m^−2^ h^−1^ at 7 atm N_2_. This pressure dependence indicates that reactant availability and mass transport can remain rate‐limiting even when efficient light harvesting is achieved [129]. Moderately textured, hierarchical or wettability‐controlled surfaces may be more suitable for sustained PEC operation than excessively dense nanostructures. Accordingly, future b‐Si‐based PEC architectures should facilitate continuous reactant supply, rapid product removal and efficient bubble detachment to maintain stable electrolyte contact and minimize local transport limitations during prolonged operation.

### 4.4 Selectivity and Side‐Reaction Control in Multi‐Electron Processes

Reaction selectivity is particularly challenging for multielectron reduction reactions such as NO_3_RR, NRR, and CO_2_RR. These reactions involve multiple adsorbed intermediates and branching pathways. They also compete strongly with proton reduction. Although the enlarged surface area of b‐Si can increase catalytic site density, it may also promote parasitic HER if the interface is not properly controlled. High photocurrent therefore does not necessarily indicate efficient solar‐fuel formation. The importance of interfacial selectivity control is evident from the 2‐ABT‐functionalized Ag/b‐Si photocathode, where molecular modification increased the CO Faradaic efficiency from approximately 50%–75% [136]. This example shows that high photocarrier generation alone is insufficient and that the local catalyst/electrolyte environment strongly governs product selectivity. Accordingly, performance assessment for these reactions should not rely on total photocurrent alone, but should also consider the Faradaic efficiency and partial current density toward the desired product. Selective conversion requires regulation of catalyst composition, local pH, reactant supply, proton availability, and intermediate binding strength [30, 31, 133, 134]. These factors are strongly coupled at the semiconductor–catalyst–electrolyte interface, indicating that selectivity control requires coordinated optimization of the catalyst and local reaction environment rather than modification of the solar absorber alone. This issue is especially severe for PEC‐NRR due to the kinetic difficulty of N_2_ activation. It is also critical for PEC‐CO_2_RR, where *COOH, *OCHO, *CO, and *CHO intermediates determine whether CO, formate, methane or other reduced products are formed. For this reason, b‐Si should be treated as a tunable photoelectrode scaffold rather than a stand‐alone solution for selectivity control. Accordingly, its optical and charge‐collection advantages must be coupled with catalysts and interfacial environments that selectively stabilize desired intermediates while suppressing competing pathways.

### 4.5 Intrinsic Photovoltage Limitation of Si

Beyond the chemical stability, catalyst integration, mass transport and selectivity‐related limitations discussed above, b‐Si is also constrained by a more fundamental limitation that originates from the intrinsic electronic properties of Si itself. Although surface texturing can dramatically suppress reflectance and enhance broadband light absorption, it does not alter the bandgap or the maximum photovoltage that can be generated by the underlying Si absorber. The bandgap of crystalline Si is approximately 1.12 eV, and the practically attainable photovoltage of a single Si photoelectrode is generally insufficient to drive unassisted overall water splitting, which requires a minimum thermodynamic potential of 1.23 V in addition to kinetic overpotentials and other voltage losses. Therefore, the optical advantages of b‐Si cannot by themselves overcome the fundamental voltage deficit of Si. This limitation becomes particularly important for bias‐free solar‐fuel production, where sufficient photovoltage must be generated within the integrated photoelectrochemical system.

Several system‐level strategies have been employed to address this limitation. Buried p–n or p–i–n junctions can increase the attainable photovoltage and improve photogenerated carrier separation by introducing an internal electric field. Tandem‐cell configurations provide another route by coupling Si with a complementary wider‐bandgap photoabsorber, allowing the photovoltages generated by the individual absorbers to be combined and thereby providing a larger overall driving force for the target reaction. As discussed in the preceding sections, buried‐junction b‐Si photocathodes and tandem b‐Si‐based PEC systems have already demonstrated the effectiveness of these approaches for improving charge separation and enabling or approaching bias‐free operation. These strategies indicate that the intrinsic voltage limitation of Si can be mitigated, but only through additional junction and device engineering beyond surface texturing alone.

However, these improvements introduce a second challenge at the system level. Buried junctions, tandem absorbers, protective layers, catalysts and additional interfaces increase the number of fabrication steps and impose stricter requirements for band alignment, current matching, interfacial stability, and large‐area process control. These factors can substantially increase manufacturing complexity and cost. This issue is particularly important for b‐Si because one of its principal advantages is the use of earth‐abundant and relatively low‐cost Si. If overcoming the photovoltage deficit requires increasingly complex multi‐material architectures, the economic benefit associated with Si may be progressively diminished. Therefore, a major challenge for commercialization is not only to achieve sufficient operating voltage but also to do so with a system architecture that preserves the low‐cost, scalable and manufacturable advantages of Si.

In summary, b‐Si remains a promising light‐harvesting platform for PEC solar‐fuel generation, but its practical implementation requires simultaneous consideration of multiple coupled limitations. As shown in Figure 10, these include chemical and electrochemical instability, the trade‐off between catalyst loading and light trapping, mass‐transport and bubble‐management constraints, selectivity losses in multielectron reactions and the intrinsic photovoltage limitation of Si. Addressing these complex challenges requires coordinated control of surface morphology, protection layers, catalyst distribution, reaction environments, junction design and system architecture. Importantly, improvements in electrochemical performance should not come at the expense of excessive structural complexity or manufacturing cost. The practical value of b‐Si will therefore depend on whether its optical advantages can be translated into stable, selective, and sufficiently photovoltage‐generating PEC systems while preserving the low‐cost and scalable attributes of Si.

**FIGURE 10:**
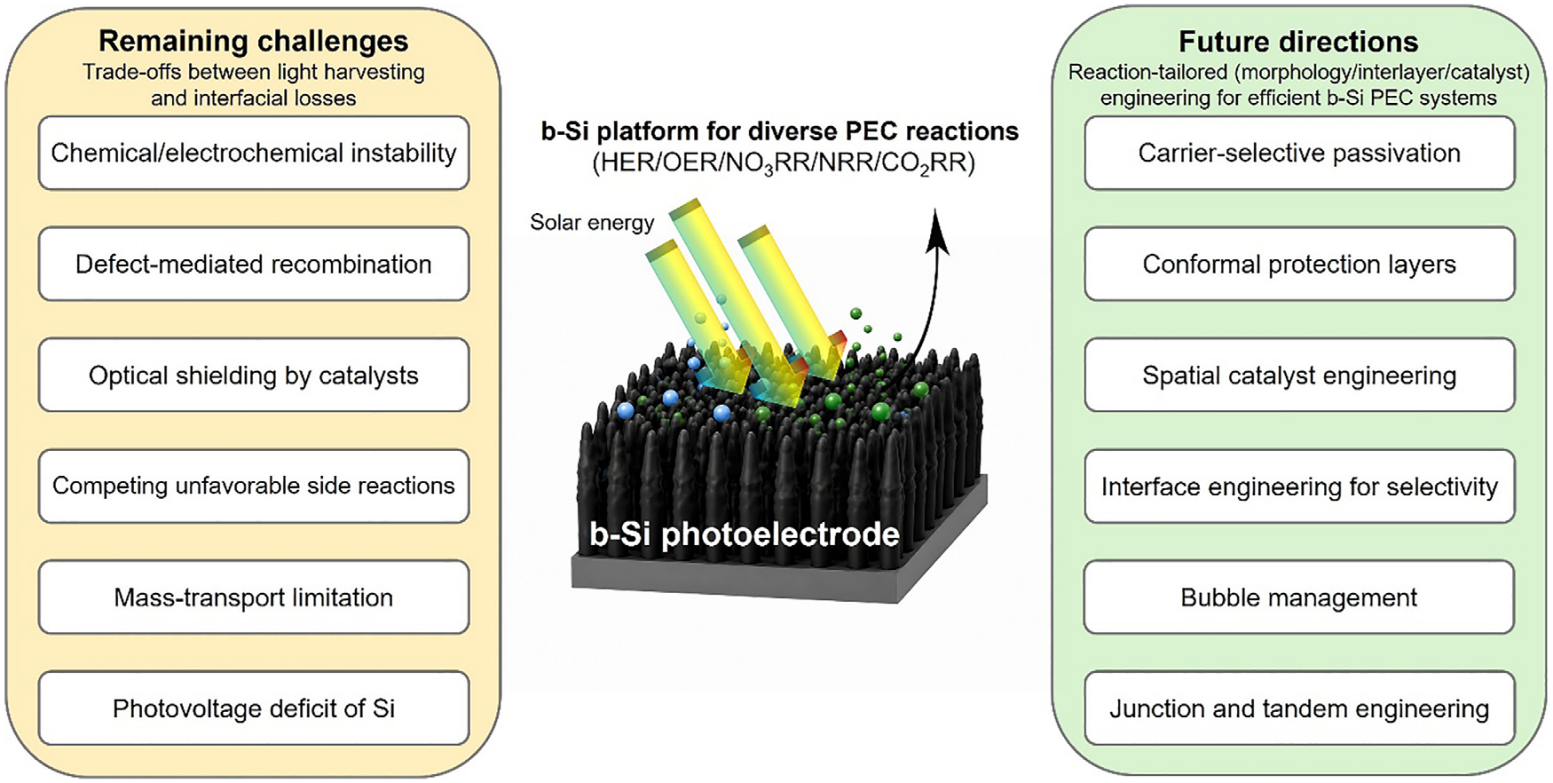
Remaining challenges and future research directions for b‐Si‐based photoelectrodes for photoelectrochemical solar fuel generation.

## 5 Conclusions and Perspectives

Silicon has long been attractive for solar energy conversion because of its suitable bandgap, earth abundance, relatively benign nature and mature processing infrastructure. Building on these intrinsic merits, b‐Si has been extensively explored as a promising photoelectrode platform for photoelectrochemical solar fuel generation. Its micro‐/nanostructured surface enables ultralow reflectance, enhanced broadband light absorption, enlarged interfacial area and abundant structural space for catalyst integration. These characteristics make b‐Si attractive for various PEC reactions including HER, OER, NO_3_RR, NRR and CO_2_RR. Despite these advantages, the performance of b‐Si photoelectrodes cannot be determined by light absorption alone. The highly textured surface can also introduce surface defects, carrier recombination, corrosion pathways and mass transport limitations. Therefore, future development should move beyond simple morphology optimization toward integrated photoelectrode design. In particular, optical light management, junction engineering, surface passivation, catalyst integration, and interfacial reaction environments must be considered together.

The requirements for b‐Si‐based PEC systems are also strongly dependent on the target reaction. Water splitting mainly requires efficient charge separation, catalytic charge transfer, and long‐term stability under reductive or oxidative conditions. By contrast, NO_3_RR, NRR, and CO_2_RR require additional control over reactant supply, intermediate stabilization, proton transfer, competing reactions and product selectivity. Thus, b‐Si should be regarded not as a universal photoelectrode structure but as a tunable platform whose morphology and interface must be adapted to each reaction pathway. Future studies should also establish distinct structure–property–performance relationships. In many reported systems, improved PEC performance can originate from enhanced photon absorption, larger catalytic areas, improved carrier extraction, suppressed recombination or altered local mass transport. Distinguishing these contributions will require systematic optical, electrochemical, spectroscopic and operando analyses [21, 145]. Such mechanistic understanding will be essential for the rational design of efficient and selective b‐Si photoelectrodes.

For practical implementation, scalable fabrication, low defect density, conformal protection, stable catalyst coupling and long‐term operation under realistic PEC conditions remain crucial. Wet etching methods offer simplicity and cost advantages, whereas dry etching and laser‐based methods provide more precise morphology control. However, each route still involves trade‐offs in uniformity, surface damage, contamination, cost, and environmental burden. Future progress will therefore depend on balancing fabrication practicality with photoelectrode performance and durability. Overall, b‐Si offers a versatile Si‐based platform for PEC solar fuel generation by integrating light harvesting, interfacial engineering, and catalytic functionality. Its future impact will rely on reaction‐adaptive design rather than the pursuit of maximum surface roughness or minimum reflectance alone. Through the coordinated optimization of morphology, passivation, catalyst interfaces and reaction environments, b‐Si‐based PEC systems can be further advanced toward efficient, durable, and selective solar fuel production.

## Author Contributions

**Neul Ha**: writing – original draft, conceptualization. **Kaiying Wang**: writing – review and editing, supervision. **Wooseok Yang**: writing – review and editing, supervision.

## Conflicts of Interest

The authors declare no conflicts of interest.

## Data Availability

The data that support the findings of this study are available from the corresponding author upon reasonable request.
